# Use and Appreciation of a Web-Based, Computer-Tailored Diet and Physical Activity Intervention Based on the Self-determination Theory: Evaluation Study of Process and Predictors

**DOI:** 10.2196/22390

**Published:** 2021-12-02

**Authors:** Juul M J Coumans, Anke Oenema, Catherine A W Bolman, Lilian Lechner

**Affiliations:** 1 Department of Health Psychology Faculty of Psychology Open University of the Netherlands Heerlen Netherlands; 2 Department of Health Promotion Faculty of Health Medicine and Life Sciences Maastricht University Maastricht Netherlands; 3 Care and Public Health Research Institute Faculty of Health Medicine and Life Sciences Maastricht University Maastricht Netherlands

**Keywords:** diet, physical activity, eHealth, self-determination theory, motivational interviewing, process evaluation, nonusage attrition

## Abstract

**Background:**

eHealth is a promising tool for promoting lifestyle behaviors such as a healthy diet and physical activity (PA). However, making people use interventions is a crucial and challenging problem in eHealth. More insight into use patterns and predicting factors is needed to improve future interventions.

**Objective:**

This study aims to examine the use, predictors of use, and appreciation of a web-based, computer-tailored, dietary and PA promotion intervention, *MyLifestyleCoach*, which is based on the self-determination theory. First, we depict the participants’ flow in the intervention and identify moments when they are likely to discontinue use. Second, we investigate whether demographic, motivational, and program-related characteristics predict the use of several intervention elements. Finally, we report the appreciation scores for the intervention and the participant and program characteristics associated with these scores.

**Methods:**

This study was based on data from web-based self-report questionnaires. Here, objectively assessed intervention use data were analyzed from participants randomized to the intervention condition. Multiple stepwise (logistic) regression analyses were conducted to examine the predictors of intervention use and evaluation scores.

**Results:**

Our findings indicate a low full completion rate for the intervention among those who chose and completed the diet module (49/146, 33.6%), the PA module (2/12, 17%), and both modules (58/273, 21.2%). Several points in the intervention where participants were likely to stop using the intervention were identified. Autonomous and intrinsic motivation toward diet were related to the completion of the initial sessions of the intervention (ie, the opening session in which participants could choose which module to follow and the first session of the diet module). In contrast, controlled motivation was linked to the completion of both modules (initial and follow-up sessions). Appreciation scores were somewhat positive. Appreciation was predicted by several motivational constructs, such as amotivation and basic psychological needs (eg, competence) and program-related features (eg, number of completed sessions).

**Conclusions:**

This study adds meaningful information on the use and appreciation of a web-based, computer-tailored dietary and PA intervention, *MyLifestyleCoach*. The results indicate that different types of motivations, such as extrinsic and intrinsic motivation, are at play at the points when people are likely to stop using the intervention. The intervention was appreciated fairly well, and several motivational constructs and fulfillment of basic psychological needs were associated with appreciation. Practical implications of these findings have been provided in this study.

## Introduction

### Background

Personalized eHealth interventions are promising for promoting a wide array of healthy lifestyle behaviors, such as physical activity (PA) and a healthy diet [1]. The true potential of such an intervention can only be reached when people are sufficiently exposed to its content [2]. However, many people do not use interventions as intended, and many people stop using the intervention before it is fully completed. Eysenbach [3] referred to this phenomenon as nonusage attrition. Research has shown that approximately 50% of the participants used a typical eHealth intervention as intended [4]. There is a general belief regarding the features that make personalized eHealth interventions effective and increase their use. The most essential elements are an increased interaction with a counselor, more frequent intended use, more frequent updates, and more extensive use of dialog support [4]. So far, a detailed understanding is lacking regarding the characteristics of participants who use an intervention as intended, how people navigate through interventions, and where they are likely to stop using the intervention.

To date, several studies have identified predictors of eHealth intervention use. In general, these studies show that age, gender, employment status, a healthier BMI, and lifestyle have been linked to the start, visit and revisit, and use of web-based interventions [5-14]. Mixed results have been found regarding marital status, working status, educational level (although numerous studies show more use for higher-educated people), income, motivation, and self-efficacy as correlates of intervention use [14]. Not only do demographic characteristics and current (lifestyle) behavior explain variance in use but also user engagement, intervention characteristics, and psychological variables could also determine an intervention’s use. Motivation toward a healthier lifestyle could be a crucial factor in use, as it has been related to the initiation and maintenance of health behavior [15,16]. Furthermore, a study found that users who were more autonomously motivated to eat healthily were less likely to stop using the intervention within the first 2 weeks of the program [17]. However, the role of motivation in use has not yet been closely examined [14].

Self-determination theory (SDT) is a macrotheory of human motivation [18]. The SDT postulates that 3 basic psychological needs must be satisfied to maintain optimal performance and well-being. These 3 basic psychological needs are autonomy, competence, and relatedness. When these needs are met, more self-determined forms of motivation are fostered, leading to more engagement in actions to achieve the intended behavior change [18-21]. In the context of use, designing an intervention in which conditions are implemented to support the basic psychological needs may also enhance participation within an intervention. For example, the basic psychological need of autonomy can be implemented in an intervention by giving participants the option to choose what parts of the intervention they want to use and when they want to start with these parts or giving participants a choice on which behavior to work. One study found several characteristics, such as current lifestyle behavior, program features, and amotivation to engage in sufficient PA, to be related to module choice within a multiple health behavior intervention [22].

So far, little is known about use and factors related to use within complex multiple-component (lifestyle) interventions, although this knowledge is very valuable for intervention improvement, particularly concerning use. An intervention that could provide useful information for this purpose is MyLifestyleCoach, a web-based, computer-tailored intervention promoting dietary and PA behavior based on SDT and motivational interviewing. This approach could be one of the underlying mechanisms of intervention use and its effectiveness. In this intervention, people can choose their own way of working through the intervention, that is, which module they want to use (ie, diet, PA, both modules, or no module), and they can decide when to start with the chosen module or modules [23,24]. This approach gives participants autonomy in selecting the behavior they prioritize at a particular moment, which is considered to increase intervention engagement and, ultimately, lower attrition and increase use. Even for people who are already (intrinsically) motivated, this intervention offers tools, such as an action plan, to turn their desire to change behavior into action. In addition to evaluating the use of MyLifestyleCoach, it is important to understand how users appreciate this intervention and whether specific characteristics predict use and appreciation.

### Objectives

The first aim of this study is to describe use of the intervention. The second aim is to examine which characteristics are linked to the use of initial and follow-up sessions. The third aim is to examine the appreciation scores for the intervention and what characteristics, especially basic psychological needs, are associated with this appreciation. This study does not shed light on the intervention’s effects; instead, this study provides useful insights for developing future eHealth interventions. For example, it gives a more in-depth understanding of whether providing participants a choice, such as which module to follow and when to follow a module, is beneficial for intervention use.

## Methods

### Study Design

A 2-group randomized controlled trial (RCT) was conducted in the Netherlands. For this study, observational data of the intervention group of this RCT, called *MyLifestyleCoach*, was used. Therefore, the control group data were excluded. *MyLifestyleCoach* is a web-based, computer-tailored intervention that consists of a diet module (*I Eat*) to promote dietary behavior and a previously tested PA module (*I Move*) to improve PA levels in Dutch adults. Participants in this intervention could choose which of these modules they would like to take part in both modules, the diet module only, the PA module only, or no module. Detailed information about the development of the intervention and the design of the RCT, which this study is part of, can be found elsewhere [23,24]. This intervention is theoretically founded on the principles of SDT and uses practical applications of motivational interviewing. This intervention was developed using the intervention mapping protocol [25]. This study was reviewed and approved by the Committee for Ethics and Consent in Research of the Open University of the Netherlands (reference U2018/07266/SVW). This study was registered in the Dutch trial register (NL7333). A data processing agreement with the software developer, who acts in line with the General Data Protection Regulation, has been signed. Furthermore, data that have been exported from the software application are safely stored at the servers of the Open University in accordance with the General Data Protection Regulation.

### Participants

The target group for this trial was Dutch adults aged 18-70 years. Participants were recruited using a research panel between October 2018 and May 2019. This research panel sent possible participants an email containing some brief information about the intervention and a link to the intervention website where they could read more information about the goal, procedure, and incentives for the study. The participants’ inclusion criteria were age between 18 and 70 years, an adequate understanding of the Dutch language, and possession of a computer or tablet with access to the internet. Participants who indicated that they had already participated in previous comparable studies of our research group were excluded.

### Procedure of the Intervention

#### Recruitment

A research panel organization sent several emails to recruit participants for this study. In this email, some basic information was provided about this study. Participants could then choose to click on a link leading them to the intervention website with additional information. If participants wanted to participate, they could click on the “I want to participate” button.

#### Preliminary Assessment and Baseline Questionnaire

First, potential participants had to fill in some questions to assess the previously described inclusion and exclusion criteria of this study and had to sign informed consent. Next, participants were randomly assigned by a computer into the intervention condition or the waiting list control condition (2:1) and filled in the baseline questionnaire. Participants in the intervention condition then continued to the opening session. Participants allocated to the waiting list control condition had no access to the intervention. After the 12-month study period, that is, when they completed the 12-month questionnaire, they were given access to the intervention.

#### Opening Session

In the opening session, participants were introduced to the program and video coaches. They also received feedback on their dietary and PA behavior using a traffic light system based on the baseline questionnaire results. Participants could receive green advice, indicating that they were already adhering to the guidelines, and following the module was unnecessary; nevertheless, they could have a look at the module. Green advice was provided for diet when they ate at least 2 portions of fruit per day, 250 g of vegetables per day, and fish once a week and consumed no unhealthy snacks per day in line with the Dutch dietary guidelines [26]. For PA, green meant that they were already engaging in ≥150 minutes of moderate to vigorous PA (MVPA) per week according to the Dutch PA guidelines [27]. For diet, orange advice indicated that they were adhering to advice for at least one targeting behavior but not all (eg, consuming sufficient fish but not vegetables). For PA, orange meant that they were engaging in 120-150 minutes of MVPA per week. This cutoff point of 120 minutes of MVPA was chosen based on previous PA guidelines. It was advised to engage in at least 30 minutes of MVPA for at least 5 days per week. Thus, 120 minutes of MVPA (or 4 days of 30-minute MVPA) meant that they almost adhered to the guideline [27], and participants were advised to follow a particular module. Red advice was provided when they did not adhere to any dietary behaviors or had <120 minutes of MVPA per week. Here, participants were strongly advised to follow a particular module. Then, participants could choose whether they wanted to follow the diet or PA module, both modules, or no module. The participants who decided to start with the diet or PA module were given the option to continue to the first session of the module immediately after this opening session or at another moment (within 14 days after the opening session). Participants who decided to follow both modules had to select the module they wanted to start directly and had to choose a date within 14 days after the opening session for the other module. The participants who decided to select no module received an email giving them the option to make a module choice again 2 weeks later. More information can be found elsewhere [22].

#### Sessions Within Modules

**Figure 1:**
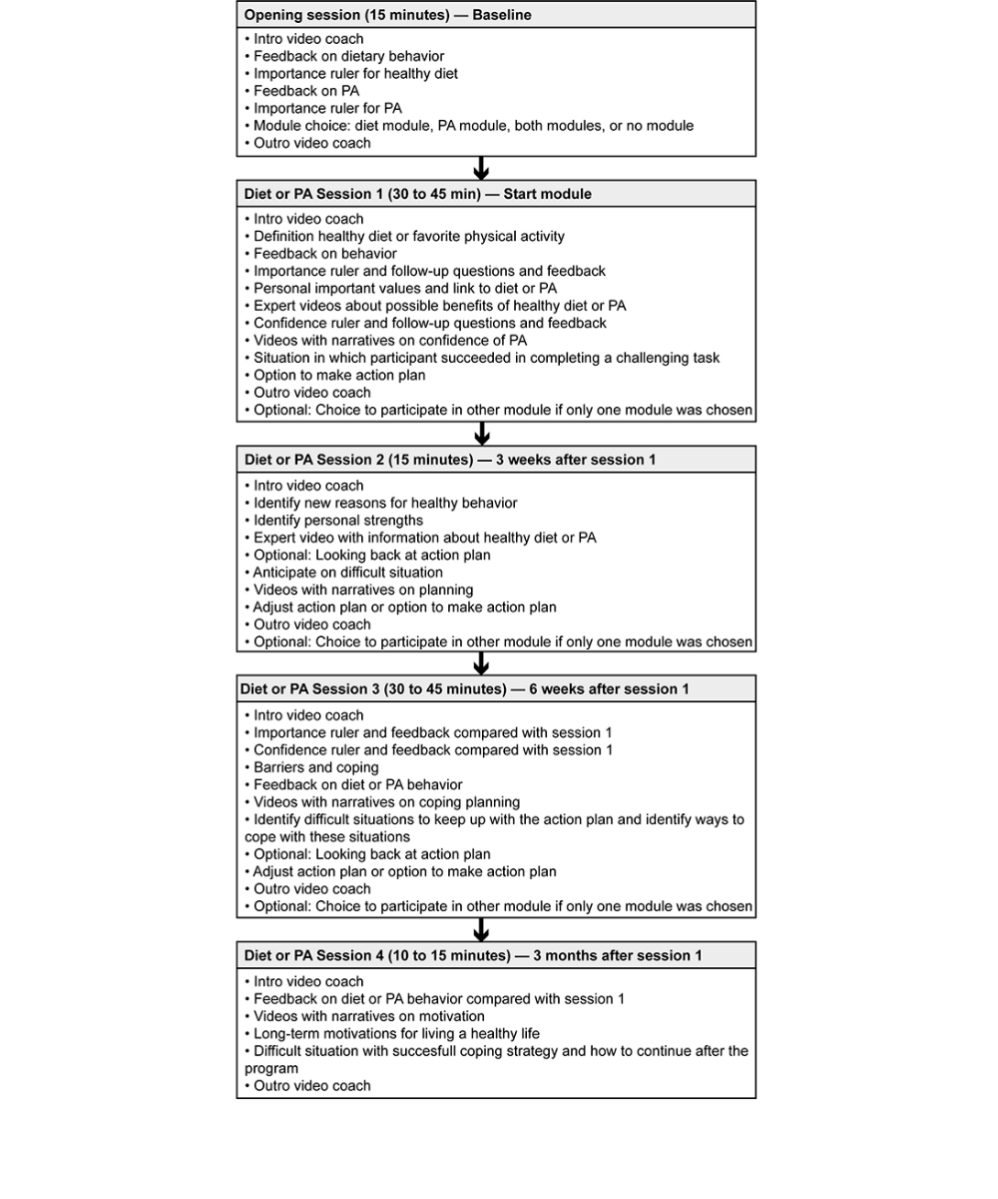
Overview of the content of the sessions in the intervention. PA: physical activity.

Both the diet and PA modules comprised 4 sessions. In *session 1,* a healthy diet was explained according to the Netherlands Nutrition Center, or guidelines for sufficient PA levels were provided. Participants were able to see their results on their dietary or PA behavior again. The importance and confidence in eating (more) healthily or engaging in sufficient PA levels were assessed, and feedback was given on this topic. Finally, participants could make an action plan. After 3 weeks from the first session, participants could enter *session 2*. In this session, they looked back on their perception of the importance of a healthy diet or PA level. They could come up with new reasons to start with the new behavior. Furthermore, they thought about what effects it would have on them if they started with the new behavior (*looking forward*). Finally, they engaged in a part on coping with difficult situations, including the identification of personal strengths, and could formulate or change the action plan. After 6 weeks from session 1, participants filled in a short questionnaire about their current behavior (diet or PA) and then entered *session 3*. An assessment took place on participants’ current perception of the importance of a healthy diet or PA and their confidence in achieving this behavior compared with session 1. Participants also received feedback on this assessment. Participants were invited to think back on a problematic situation in which they struggled but managed the achieved behavior. They received feedback on their current dietary or PA behavior compared with session 1 and could formulate or change the action plan expressed in the previous session of sessions. After 3 months from session 1, they filled in a short questionnaire regarding their current behavior (diet or PA) and then entered *session 4*, which served as a booster session. Participants could choose several topics from previous sessions that they wanted to do. These topics included feedback on their current behavior compared with session 1, long-term personal motivation and confidence, how to deal with difficult situations, and information on how to maintain their new behavior after the end of the program. Figure 1 shows an overview of the content of these sessions. More detailed information on these sessions can be found in the protocol papers [23,24].

### Follow-up Questionnaire

After 6 months from when participants completed the baseline questionnaire, both in the intervention and control conditions, they were sent an invitation email to complete the 6-month follow-up questionnaire. Email reminders were sent every week for 4 weeks in total. Participants who completed all questionnaires were entered into a draw for 2 tablets and gift vouchers of up to €50 (US $57.23).

### Measurements

The baseline questionnaire assessed demographic characteristics, dietary and PA behaviors, and psychosocial constructs. All these measurements were self-reported.

#### Demographics

Demographic characteristics included age, gender, education, work status, physical impairment, marital status, weight and height, and health status using a thermometer-style visual analog scale ranging from 0-100. These factors served as control variables in our analysis.

#### Motivation

Of the psychological constructs measured in this study, only motivation was included. Motivation was assessed with 2 Treatment Self-Regulation Questionnaires, one for dietary behavior and the other for PA behavior [28]. Participants had to indicate the degree to which they agreed with each of the 15 statements on a 7-point Likert scale. There were 3 subscales: amotivation (3 items), controlled motivation (6 items), and autonomous motivation (6 items). This questionnaire did not assess the intrinsic motivation for these health-related behaviors. For that purpose, we included the *intrinsic regulation* subscale (4 items) from the Dutch Behavioral Regulation in Exercise Questionnaire-2 to determine the intrinsic motivation for PA behavior and an adapted version of the Behavioral Regulation in Exercise Questionnaire-2 to determine the intrinsic motivation for dietary behavior [29]. Participants had to indicate the degree to which they agreed with each of the 4 statements on a 5-point Likert scale. The mean score was calculated for each motivational construct.

#### Dietary and PA Behavior

Dietary behavior was assessed using a validated Food Frequency Questionnaire. The Food Frequency Questionnaire was extended with questions regarding the size of vegetable and fruit portions based on the study by Huybrechts et al [30]. The outcomes were fruit intake, vegetable intake, fish consumption, and daily consumption frequency of unhealthy snacks. For the calculation of the consumption frequency of unhealthy snacks, we referred to the study by Coumans et al [31]. PA behavior for a typical week in the past month was assessed using the validated Dutch Short Questionnaire to Assess Health [32]. PA behavior was operationalized as the total number of minutes of MVPA by multiplying the frequency (days per week) and duration (hours and minutes per day) of leisure and transport walking, leisure and transport cycling, occupational activities, household activities, gardening, odd jobs, and sports performed with moderate or vigorous intensity.

### Process Evaluation

To assess appreciation, participants were asked to give an appreciation score for the whole program on a 10-point scale at 6 months from baseline. People also had to provide a rating for the diet and PA module, which ranged on a scale from 1 (very low) to 10 (very high), if they had completed at least 1 session of the particular module. Furthermore, participants were asked to what extent the program met their basic psychological needs during the intervention on a 5-point Likert scale from 1 (fully disagree) to 5 (fully agree) [33]. A total of 2 items assessed autonomy: (1) participants were asked if they could determine which goals they could set and (2) which information and pieces of advice they could read in the intervention. Relatedness was assessed by 3 items: (1) participants were asked if they felt involved in the intervention, (2) if the intervention was personal, and (3) if they felt supported by the intervention. Competence was assessed with 1 item: participants were asked whether they had confidence in eating (more) healthily and/or engaging in more or sufficient PA. The mean score for each of these basic psychological needs was calculated.

Finally, data on the completeness of sessions were used to determine how many participants used a specific part of the intervention (*use*). A completeness variable (1=completed and 0=not completed) was created for each session: the opening session and module sessions 1, 2, 3, and 4 of the diet and PA modules of the intervention. When participants finished a session, the completeness variable was set to 1. For this study, 5 use variables were created: (1) finished opening session (1=yes and 0=no), (2) finished the first session of the diet module when only the diet module was chosen (1=yes and 0=no), (3) finished the first sessions of both the diet and PA modules when both modules were chosen (1=yes and 0=no), (4) finished the whole diet module when only the diet module was chosen (1=yes and 0=no; based on 4 complete sessions), and (5) finished the diet and PA module when both modules were chosen (1=yes and 0=no; based on 8 complete sessions).

### Statistical Analysis

Descriptive statistics (mean and SD values) and frequencies (and percentages) were used to depict the characteristics of the participants, the overall flow through the intervention, and appreciation scores. Logistic regression analyses were conducted to examine which personal characteristics (age, gender, education, marital status, work, physical impairment, health status, and BMI) and motivational characteristics were related to use. Use was subdivided into 3 parts according to different points in the intervention: (1) completion of the opening session; (2) the initial module’s session, that is, completion of the first session of the diet module when only the diet module was chosen or the first session of the diet and PA module when both modules were chosen; and (3) the follow-up sessions, that is, completion of all 4 sessions of the diet module when only the diet module was chosen or completion of all 8 sessions of the diet and PA module when both modules were chosen. Furthermore, linear regression analyses were performed to investigate which demographic factors, motivational constructs, and program features were associated with the intervention appreciation scores (overall intervention, diet, and PA module). All statistical analyses were performed using the statistical software R (version 3.6.0; R Foundation for Statistical Computing). For all regression analyses, a stepwise approach was used in which the demographic variables were entered in the first step, motivational constructs were introduced in the second step, and program features were added in the third step. Variance inflation factors were inspected before conducting the analyses. Statistical significance was set at *P*<.05.

## Results

### Participants’ Characteristics

The mean age of the sample was 51.9 (SD 13.1) years; there were slightly more women than men participating in the study; 70.3% (545/775) of the sample was highly educated; and 64% (496/775) were employed. The mean BMI of this sample was considered to be slightly overweight. However, the proportion of participants with a healthy weight was the largest. More characteristics are presented in Table 1.

**Table 1:** Demographic characteristics of the full sample (N=775).

Variable			Value
Age (years), mean (SD)			51.9 (13.1)
**Gender, n (%)**			
	Women	475 (61.3)	
	Men	300 (38.7)	
**Education, n (%)**			
	Low	29 (3.7)	
	Medium	201 (25.9)	
	High	545 (70.3)	
**Marital status, n (%)**			
	Partner	529 (68.3)	
	Single	246 (31.7)	
**Work, n (%)**			
	Employed	496 (64.0)	
	Unemployed	279 (36.0)	
**Physical impairment, n (%)**			
	No	740 (95.5)	
	Yes	35 (4.5)	
**BMI status^a^, mean (SD)**			
	Underweight	15 (1.9)	
	Normal	328 (42.3)	
	Overweight	279 (36.0)	
	Obese	153 (19.7)	
BMI (kg/m^2^), mean (SD)			26.5 (5.2)
Health status (0-100), mean (SD)			69.9 (15.6)
Amotivation diet (1-7), mean (SD)			2.3 (1.2)
Controlled motivation diet (1-7), mean (SD)			2.8 (1.2)
Autonomous motivation diet (1-7), mean (SD)			5.5 (1.2)
Intrinsic motivation diet (1-5), mean (SD)			3.5 (1.0)
Amotivation PA^b^ (1-7), mean (SD)			2.2 (1.3)
Controlled motivation PA (1-7), mean (SD)			2.7 (1.2)
Autonomous motivation PA (1-7), mean (SD)			5.6 (1.2)
Intrinsic motivation PA (1-5), mean (SD)			3.8 (1.1)
Fruit, mean (SD)			1.4 (1.1)
Vegetables, mean (SD)			143.1 (80.7)
Fish (0-7), mean (SD)			1.1 (1.1)
Unhealthy snacks, mean (SD)			1.5 (1.9)
MVPA^c^, mean (SD)			992.7 (836.8)

^a^Underweight: a BMI value of <18.5 kg/m^2^; normal weight: a BMI value ranging from 18.5 kg/m^2^ to <25.0 kg/m^2^; overweight: a BMI value ranging from 25.0 kg/m^2^ to <30.0 kg/m^2^; and obese: a BMI value of ≥30.0 kg/m^2^.

^b^PA: physical activity.

^c^MVPA: moderate to vigorous physical activity.

### Description of the Participants’ Flow and Module Use

**Figure 2:**
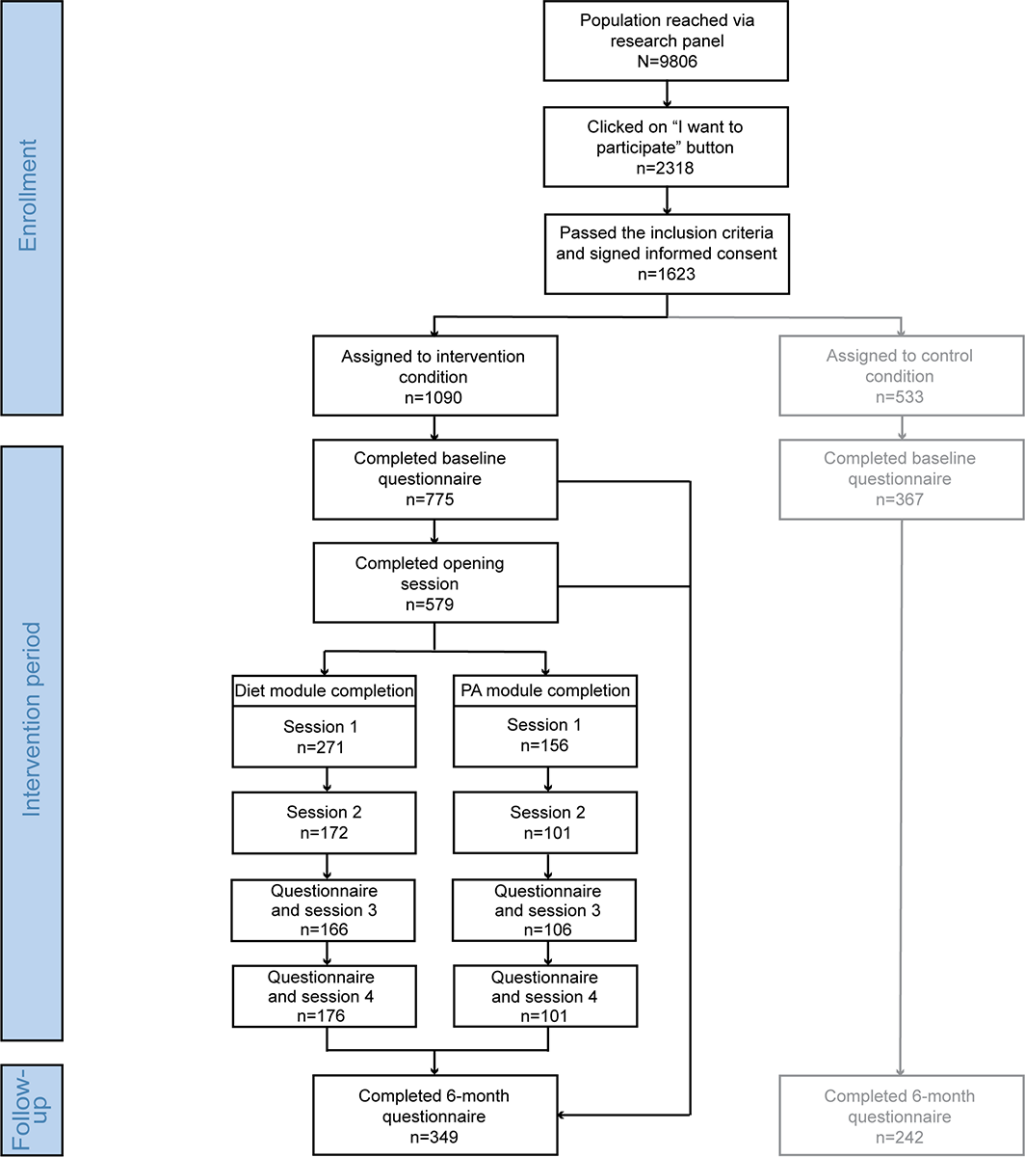
Participants' flow in the randomized controlled trial. Participants did not need to complete the second session to be able to continue the third session. Therefore, use rates do not necessarily represent a funnel shape. PA: physical activity.

Figure 2 illustrates the flow of participants. The boxes and text in gray represent the control conditions. This study focuses solely on the use and appreciation of the intervention. Therefore, the control condition was not included, as they did not take part in the intervention in this time frame. In total, 9806 individuals were directly contacted via the research panel organization. Of these, 23.64% (2318/9806) of individuals visited the study website and clicked on the “I want to participate” button; 16.55% (1623/9806) of these individuals passed the inclusion criteria and signed the informed consent and were randomized into the 2 conditions. Several individuals in the intervention condition did not complete the baseline questionnaire after randomization (315/1090, 28.9%). Of the 775 participants in the intervention condition, 619 (79.9%) made a choice on which module (diet, PA, both, or none) to follow, and 579 (74.7%) participants completed the entire opening session.

Of the 158 participants who chose to follow the diet module only, 8 (5.1%) participants did not choose whether they wanted to start immediately or later or did not fill in when to start with the first session, and 4 (2.5%) participants did not receive an invitation mail for the first session. Of the remaining 146 participants, half of them started immediately, whereas the other half wanted to start later. Of the 73 participants who decided to start immediately, 44 (60%) completed session 1, 32 (44%) completed session 2, 32 (44%) completed session 3, and 35 (48%) completed session 4. Of the 73 participants who decided to start later, 39 (53%) completed session 1, 26 (37%) completed session 2, 28 (38%) completed session 3, and 28 (38%) completed session 4. Approximately 33.6% (49/146) of participants completed all 4 sessions in the diet module.

Within the PA module (n=12, as 2 participants did not receive an invitation mail for the first session), half of the participants decided to start immediately, and the other half wanted to start later. Of the 6 participants who decided to start immediately, 4 (67%) completed session 1, 2 (33%) completed session 2, 1 (17%) completed session 3, and 1 (17%) completed the fourth session. Of the 6 participants who decided to start later, only 1 (17%) completed all 4 sessions. Participants who only chose the diet module were asked after every session, except for the last one, whether they were interested in starting with the PA module. Of the 17 participants who chose to start with the PA module, 8 (47%) completed the first session of the PA module, 7 (41%) completed the second and third sessions, and 4 (24%) completed the last session. Participants who only chose the PA module were asked after every session, except for the last one, whether they were interested in starting with the diet module. Of the 12 participants who only chose the PA module, 1 (8%) participant was interested in the diet module; however, this person did not complete any session of the diet module.

**Figure 3:**
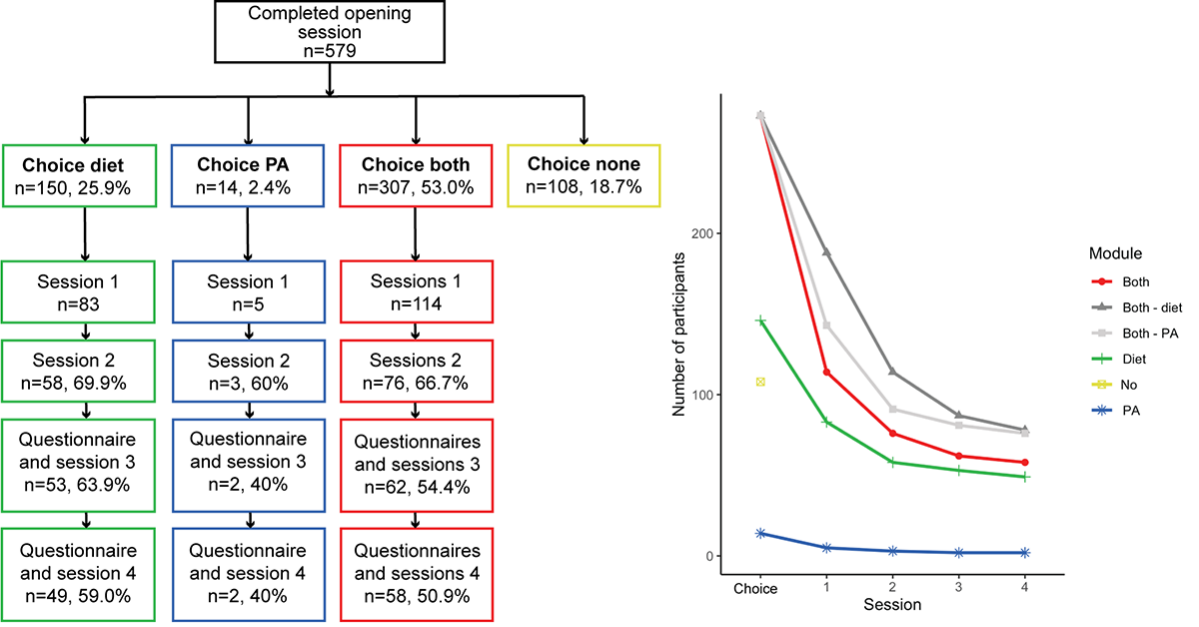
Completion rates (left panel) and curves (right panel) stratified for module choice. PA: physical activity.

Of the 339 who chose both modules, 32 (9.4%) did not complete the opening session, and 34 (10%) did not receive an invitation mail for the second module because of a technical error. Most participants decided to start with the diet module (244/273, 89.4%) compared with the PA module (29/273, 10.6%). Full completion rates, that is, those who completed all sessions as intended, can be found in Figure 3. In the right panel, 2 lines are added that represent the people who chose both modules who completed the sessions of the separate diet module (dark gray) or completed the sessions of the separate physical activity module (light gray). Approximately 21.2% (58/273) of participants completed all sessions of both modules. Approximately 5% (4/77) of participants who preferred to start later with the module did not receive an invitation email for the first session of the diet module because of a technical error. Moreover, approximately 25% (2/8) of participants who preferred to start later with the module did not receive an invitation email for the first session of the PA module because of a technical error.

Participants who initially chose no module in the intervention (110/775, 14.2%) were sent an email directing them to the website where they could change their choice. Of the 110 participants, only 4 (3.6%) reconsidered their choice, and 2 (1.8%) chose a module. Of the 110 participants, 1 (0.9%) chose the diet module, 1 (0.9%) chose the PA module (and did not finish any modules), and 2 (1.8%) chose the no module option again.

Finally, 45% (349/775) of participants completed the follow-up questionnaire at 6 months from baseline. Of the 158 participants who chose the diet module, 78 (49.4%) completed the follow-up questionnaire. Of the 14 participants who chose the PA module, 9 (64.3%) completed the follow-up questionnaire. Of the 339 participants who chose both modules, 151 (44.5%) completed the follow-up questionnaire. Of the 108 participants who did not choose any module in the opening session, 58 (53.7%) completed the follow-up questionnaire. Of the 156 participants who did not enter the opening session or made no module choice, 53 (33.9%) completed the follow-up questionnaire. This follow-up measurement included the process evaluation questions.

### Predictors of Use of Initial and Follow-up Sessions

As described in the previous section and as can be seen in the participants’ flowchart (Figure 2) and the completion rates in Figure 3, there are several moments within the intervention at which participants stop using the intervention. First, several people did not complete the baseline questionnaire after randomization (315/1090, 28.9%; Figure 2). As we did not have the demographic characteristics of this group, it was not possible to further examine predictors of why they stopped using the intervention. Second, there was a significant number of participants who did not complete the opening session after completing the baseline questionnaire (Figure 2). Third, another group did not start or end the first session of their chosen module (Figure 3). Fourth, numerous people did not complete the sessions as intended, that is, about half of the participants completed the whole intervention once started (Figure 3). Here, we investigate whether there are characteristics associated with use for the latter 3 moments.

The logistic regression analysis (Table 2) showed that participants with a partner and those who had a higher intrinsic motivation to eat healthily were less likely to complete the opening session. Participants with higher scores on autonomous motivation to eat healthily and those with a higher score on intrinsic motivation to engage in sufficient PA were more likely to complete the opening session. Therefore, intrinsic motivation toward PA increased the likelihood of completing the opening session, whereas intrinsic motivation toward a healthy diet decreased the likelihood of completing the opening session.

The logistic regression analyses relating demographic characteristics, motivational constructs, and intervention features with completing the first session showed that people who had a physical impairment and those with higher scores on autonomous motivation to eat healthily were more likely to complete the first session of the diet module. On the other hand, participants with a higher BMI or more intrinsic motivation to eat healthily were less likely to complete the first session of the diet module. For participants who chose both modules, the results showed that participants having a higher self-reported health status, more controlled motivation to PA, and receiving red advice for PA compared with orange advice were more likely to complete the first sessions of both modules. On the contrary, participants with a partner or those who preferred to start with the PA module were less likely to complete the first sessions of both modules. The results are presented in Table 3. Owing to the low number of participants that only chose the PA module, predictors of use for the PA module were not further investigated, as it would have been statistically invalid.

Finally, the regression analyses relating demographic characteristics, motivational constructs, and intervention features with completing the entire intervention are presented in Table 4. The *whole intervention* could concern the 4 sessions of (1) the diet module, (2) the PA module—owing to the low number of participants that only chose the PA module, predictors of use for the PA module were not further investigated, as it would have been statistically invalid—and (3) both the diet and PA module. For participants who only chose the diet module, it was found that those who received red advice for diet in the opening session compared with orange advice were more likely to complete all sessions within the diet module. For participants who chose both modules, the results showed that older participants and those with a higher controlled motivation toward PA were more likely to complete all sessions of both modules. On the other hand, participants with more controlled motivation toward diet, those receiving red advice for diet compared with orange advice, or those who preferred to start with the PA module were less likely to complete all sessions of both modules. The full basic models can be found in Multimedia Appendix 1.

**Table 2:** Results of the stepwise logistic regression analyses (full model) showing variables associated with completing the opening session (N=775)^a^.

Predictors	Completed opening session (1=yes and 0=no)	
	Odds ratio (95% CI; SE)	*P* value
Intercept	1.31 (0.21-8.09; 0.93)	.77
Age	1.003 (0.99-1.02; 0.01)	.69
Gender^b^	1.27 (0.88-1.84; 0.19)	.20
Education high^c^	0.98 (0.66-1.47; 0.21)	.93
Education low^c^	1.09 (0.40-2.94; 0.51)	.86
Marital status partner^d^	0.60 (0.41-0.89; 0.20)	*.*01^e^
Work employed^f^	0.97 (0.67-1.41; 0.19)	.87
Impairment^g^	1.74 (0.66-4.57; 0.49)	.26
BMI	1.04 (0.996-1.08; 0.02)	.08
Health status	1.0005 (0.99-1.01; 0.01)	.94
Amotivation diet	1.09 (0.88-1.33; 0.10)	.43
Amotivation PA^h^	0.90 (0.74-1.10; 0.10)	.30
Controlled motivation diet	0.95 (0.75-1.20; 0.12)	.68
Controlled motivation PA	1.12 (0.89-1.42; 0.12)	.33
Autonomous motivation diet	1.35 (1.05-1.74; 0.13)	.02^e^
Autonomous motivation PA	0.80 (0.61-1.04; 0.13)	.10
Intrinsic motivation diet	0.60 (0.48-0.76; 0.12)	<.001^e^
Intrinsic motivation PA	1.38 (1.13-1.68; 0.10)	.001^e^

^a^The results’ interpretations are reported when all other predictors are held constant. Explained variance *R*^2^ tjur=0.054; Akaike information criterion=869.59.

^b^*Female* is the reference category.

^c^*Medium education* is the reference category.

^d^*Single* is the reference category.

^e^Values represent statistical significance.

^f^*Being unemployed* is the reference category.

^g^*No physical impairment* is the reference category.

^h^PA: physical activity.

**Table 3:** Results of the stepwise logistic regression analyses (full model) showing variables associated with completing the first session for the diet module and both modules.

Predictors	Session 1 (1=yes and 0=no)			
	Diet module^a,b^		Both modules^c^	
	OR^d^ (95% CI; SE)	*P* value	OR (95% CI; SE)	*P* value
Intercept	2.88 (0.04-221.08; 2.22)	.63	0.06 (0.002-2.39; 1.85)	.14
Age	1.02 (0.99-1.06; 0.02)	.26	1.02 (0.99-1.04; 0.01)	.15
Gender^e^	0.69 (0.31-1.56; 0.41)	.38	0.70 (0.38-1.31; 0.32)	.27
Education high^f^	1.02 (0.38-2.73; 0.50)	.96	1.39 (0.73-2.66; 0.33)	.32
Education low^f^	1.53 (0.19-12.44; 1.07)	.69	1.21 (0.25-5.91; 0.81)	.82
Marital status partner^g^	1.03 (0.43-2.45; 0.44)	.95	0.52 (0.28-0.95; 0.31)	.03^h^
Work employed^i^	1.26 (0.53-3.01; 0.44)	.60	0.84 (0.46-1.54; 0.31)	.57
Impairment^j^	9.05 (1.06-77.10; 1.09)	.04^h^	3.04 (0.79-11.75; 0.69)	.12
BMI	0.87 (0.79-0.97; 0.05)	.009^h^	0.98 (0.93-1.04; 0.03)	.55
Health status	1.02 (0.99-1.05; 0.02)	.25	1.04 (1.01-1.06; 0.01)	.001^h^
Amotivation diet	0.83 (0.49-1.39; 0.26)	.47	1.19 (0.84-1.67; 0.17)	.33
Amotivation PA^k^	1.50 (0.95-2.37; 0.23)	.08	0.72 (0.49-1.06; 0.20)	.10
Controlled motivation diet	1.24 (0.68-2.25; 0.30)	.48	0.70 (0.47-1.06; 0.21)	.09
Controlled motivation PA	0.65 (0.37-1.15; 0.29)	.14	1.70 (1.13-2.57; 0.21)	.01^h^
Autonomous motivation diet	2.27 (1.17-4.41; 0.34)	.02^h^	0.995 (0.64-1.55; 0.23)	.98
Autonomous motivation PA	0.69 (0.36-1.33; 0.33)	.27	0.97 (0.61-1.55; 0.24)	.90
Intrinsic motivation diet	0.53 (0.31-0.91; 0.27)	.02^h^	0.94 (0.66-1.33; 0.18)	.71
Intrinsic motivation PA	1.05 (0.65-1.33; 0.25)	.83	0.80 (0.58-1.11; 0.17)	.19
Diet advice green^l,m^	—^n^	—	0.00 (974.90)	.99
Diet advice red^l^	2.77 (0.98-7.86; 0.53)	.06	0.59 (0.31-1.12; 0.33)	.11
PA advice green^l^	—	—	3.18 (0.40-24.92; 1.05)	.27
PA advice red^l^	—	—	16.82 (1.28-221.17; 1.31)	.03^h^
Module start (later^o^)	0.55 (0.25-1.22; 0.40)	.14	—	—
First module (PA^p^)	—	—	0.21 (0.07-0.63; 0.56)	.005^h^

^a^Physical activity advice was not included, as 3 participants did not receive green advice. Odds ratios were unreliable when this variable was included in the analyses. The results’ interpretations are reported when all other predictors are held constant.

^b^Observations=146; *R*^2^ tjur=0.158; Akaike information criterion=215.19.

^c^Observations=273; *R*^2^ tjur=0.167; Akaike information criterion=366.99.

^d^OR: odds ratio.

^e^*Female* is the reference category.

^f^*Medium* education is the reference category.

^g^*Single* is the reference category.

^h^Values represent statistical significance.

^i^*Being* unemployed is the reference category.

^j^*No physical impairment* is the reference category.

^k^PA: physical activity.

^l^*Orange advice* is the reference category.

^m^Only 2 participants received green advice. Consequently, the odds ratio and SE are less reliable, and CI is not reported.

^n^These variables were not included in the model.

^o^*Directly starting with the first session* was the reference category.

^p^*Choosing the diet module to start with when both modules were chosen* was the reference category.

**Table 4:** Results of the stepwise logistic regression analyses (full model) showing variables associated with completing all sessions when the diet or both modules were chosen^a^.

Predictors	All sessions (1=yes and 0=no)			
	Diet module^b^		Both modules^c^	
	OR^d^ (95% CI; SE)	*P* value	OR (95% CI; SE)	*P* value
Intercept	0.05 (0.001-3.91; 2.25)	.18	0.22 (0.003-14.94; 2.16)	.48
Age	1.04 (0.998-1.08; 0.02)	.07	1.03 (1.002-1.07; 0.02)	.04^e^
Gender^f^	0.99 (0.44-2.22; 0.41)	.98	1.08 (0.52-2.25; 0.37)	.83
Education high^g^	1.55 (0.60-4.04; 0.49)	.37	1.34 (0.61-2.96; 0.40)	.47
Education low^g^	1.32 (0.17-10.57; 1.06)	.79	1.12 (0.15-8.10; 1.01)	.91
Marital status partner^h^	0.91 (0.38-2.18; 0.44)	.84	0.51 (0.25-1.04; 0.37)	.06
Work employed^i^	0.85 (0.35-2.07; 0.45)	.72	0.73 (0.36-1.49; 0.36)	.39
Impairment^j^	0.88 (0.14-5.32; 0.92)	.89	1.82 (0.36-9.13; 0.82)	.47
BMI	0.95 (0.86-1.05; 0.05)	.32	0.95 (0.89-1.02; 0.03)	.14
Health status	1.01 (0.98-1.04; 0.02)	.63	1.02 (0.99-1.04; 0.01)	.17
Amotivation diet	1.19 (0.71-1.99; 0.26)	.51	1.27 (0.85-1.89; 0.20)	.25
Amotivation PA^k^	1.19 (0.76-1.85; 0.23)	.45	0.95 (0.60-1.49; 0.23)	.82
Controlled motivation diet	1.09 (0.60-1.97; 0.30)	.79	0.43 (0.25-0.75; 0.28)	.003^e^
Controlled motivation PA	0.63 (0.35-1.13; 0.30)	.12	2.60 (1.48-4.59; 0.29)	.001^e^
Autonomous motivation diet	1.55 (0.82-2.95; 0.33)	.18	0.92 (0.52-1.53; 0.25)	.74
Autonomous motivation PA	0.82 (0.44-1.53; 0.32)	.52	0.89 (0.52-1.53; 0.28)	.67
Intrinsic motivation Diet	0.96 (0.56-1.64; 0.28)	.88	0.83 (0.53-1.30; 0.23)	.42
Intrinsic motivation PA	1.07 (0.65-1.75; 0.25)	.79	0.92 (0.62-1.39; 0.21)	.70
Diet advice green^l,m^	—^n^	—	0.00 (951.69)	.99
Diet advice red^l^	3.17 (1.11-9.04; 0.53)	.03^e^	0.39 (0.16-0.90; 0.43)	.03^e^
PA advice green^l^	—	—	1.69 (0.15-19.40; 1.24)	.67
PA advice red^l^	—	—	9.89 (0.50-194.10; 1.52)	.13
Start (later^o^)	0.56 (0.26-1.23; 0.40)	.15	—	—
First module (PA^p^)	—	—	0.05 (0.01-0.50; 1.16)	.01^e^

^a^The results’ interpretations are reported when all other predictors are held constant.

^b^Observations=146; *R*^2^ tjur=0.099; Akaike information criterion=211.14.

^c^Observations=273; *R*^2^ tjur=0.188; Akaike information criterion=276.69.

^d^OR: odds ratio.

^e^Values represent statistical significance.

^f^*Female* is the reference category.

^g^*Medium education* is the reference category.

^h^*Single* is the reference category.

^i^*Being unemployed* is the reference category.

^j^*No physical impairment* is the reference category.

^k^PA: physical activity.

^l^*Orange advice* is the reference category.

^m^Only 2 participants received green advice. Consequently, the odds ratio and SE are less reliable, and CI is not reported.

^n^These variables were not included in the model.

^o^*Directly starting with the first session* was the reference category.

^p^*Choosing the diet module to start with when both modules were chosen* was the reference category.

### Appreciation and Its Predictors

After 6 months from baseline, participants were asked to complete the follow-up questionnaire, including the process evaluation measures. These process evaluation measurements focused on the extent to which the program met the participants’ basic psychological needs, which were operationalized as the ratings they gave for autonomy, competence, and relatedness. The mean appreciation score for the intervention as a whole was 6.9 (SD 1.7). Approximately 83.3% (245/294) of participants provided a rating of 6 out of 10 or higher (*sufficient*). Overall, the mean scores of the process evaluation variables represented neutral (relatedness and competence) to positive scores (autonomy) of the intervention. For autonomy, the average rating was 3.9 (SD 0.8; 291/294, 99%) out of 5. For relatedness, the average rating was 3.1 (SD 0.9; 291/294, 99%) out of 5. For competence, the average rating was 3.0 (SD 1.0; 291/294, 99%) out of 5. The appreciation of the diet module was, on average, 7.1 (SD 1.7; 159/294, 54.1%), whereas the appreciation of the PA module was, on average, 7.4 (SD 1.7; 101/294, 34.4%).

The results of the regression analyses with appreciation scores can be found in Table 5. The results showed that there were no demographic characteristics associated with appreciation scores. The only variable that was significantly associated with all appreciation scores was competence: feeling more confident because of the program in eating (more) healthily or engaging in sufficient PA was associated with higher appreciation scores. For the overall appreciation score, it was found that choosing both modules compared with no module was linked to a lower appreciation score. However, completing more sessions in the PA module was related to a higher appreciation score. For both the diet and PA appreciation scores, the results showed that feeling more related to the program was linked to higher appreciation scores. For the appreciation of the diet module, it was found that a higher amotivation to PA and being more intrinsically motivated to eat (more) healthily was linked to higher appreciation scores. For the appreciation of the PA module, it was found that a higher amotivation to eat (more) healthily was related to a lower appreciation score, whereas being more autonomously motivated to engage in sufficient PA was linked to a higher appreciation score. The full basic models can be found in Multimedia Appendix 2.

**Table 5:** Results of the stepwise regression analyses (full model) showing variables associated with appreciation scores^a^.

Predictors	Appreciation^b^				Appreciation of diet module^c^				Appreciation of PA^d^ module^e^		
	b^f^ (SE)	B^g^	*P* value	b (SE)		B	*P* value	b (SE)		B	*P* value
Intercept	0.43 (1.17)	0.00	.71	1.17 (1.35)		0.00	.39	0.36 (1.63)		0.00	.83
Age (years)	0.01 (0.01)	0.06	.35	−0.00 (0.01)		−0.03	.71	−0.01 (0.01)		−0.05	.57
Gender^h^	−0.11 (0.18)	−0.03	.56	−0.15 (0.20)		−0.04	.45	0.19 (0.27)		0.05	.49
Education high^i^	0.14 (0.21)	0.04	.50	0.05 (0.22)		0.01	.81	−0.05 (0.28)		−0.01	.85
Education low^i^	0.28 (0.46)	0.03	.54	−0.25 (0.43)		−0.03	.57	0.14 (0.71)		0.01	.85
Marital partner^j^	0.21 (0.19)	0.06	.29	0.03 (0.21)		0.01	.88	0.04 (0.29)		0.01	.89
Work^k^	0.0003 (0.19)	0.0001	.99	0.07 (0.20)		0.02	.71	0.05 (0.28)		0.01	.86
Impairment^l^	−0.73 (0.41)	−0.10	.08	−0.09 (0.39)		−0.01	.82	−0.17 (0.49)		−0.03	.72
BMI	0.01 (0.02)	0.03	.55	−0.01 (0.02)		−0.04	.59	−0.02 (0.02)		−0.09	.26
Health status	0.006 (0.01)	0.04	.52	−0.002 (0.01)		−0.02	.80	−0.01 (0.01)		−0.05	.56
Amotivation diet	0.14 (0.11)	0.10	.20	−0.10 (0.11)		−0.07	.38	−0.37 (0.18)		−0.24	.05^n^
Amotivation PA	0.0003 (0.11)	0.0002	.99	0.24 (0.12)		0.17	.05^n^	0.34 (0.17)		0.23	.06
Controlled motivation Diet	0.07 (0.12)	0.05	.56	−0.09 (0.15)		−0.07	.53	0.02 (0.17)		0.02	.90
Controlled motivation PA	−0.14 (0.12)	−0.10	.27	−0.09 (0.14)		−0.07	.51	−0.15 (0.17)		−0.11	.41
Autonomous motivation diet	−0.05 (0.14)	−0.03	.74	0.25 (0.19)		0.17	.18	−0.10 (0.19)		−0.07	.61
Autonomous motivation PA	0.23 (0.14)	0.15	.09	0.09 (0.19)		0.06	.64	0.52 (0.20)		0.37	.01^n^
Intrinsic motivation diet	0.19 (0.12)	0.10	.11	0.31 (0.13)		0.18	.02^n^	0.12 (0.18)		0.07	.51
Intrinsic motivation PA	−0.12 (0.10)	−0.08	.23	−0.09 (0.11)		−0.06	.40	−0.04 (0.15)		−0.03	.82
Autonomy	0.22 (0.13)	0.10	.10	0.17 (0.15)		0.06	.27	0.32 (0.21)		0.12	.13
Relatedness	0.31 (0.18)	0.16	.10	0.38 (0.17)		0.20	.03^m^	0.51 (0.25)		0.26	.05^m^
Competence	0.59 (0.14)	0.34	<.001^m^	0.78 (0.13)		0.49	<.001^m^	0.52 (0.18)		0.34	.005^m^
Diet advice green^n^	1.25 (1.43)	0.04	.38	—^o^		—	—	—		—	—
Diet advice red^n^	0.14 (0.21)	0.03	.49	−0.07 (0.22)		−0.02	.74	−0.22 (0.29)		−0.05	.45
PA advice green^n^	0.43 (0.61)	0.06	.48	0.04 (0.68)		0.01	.96	0.44 (0.77)		0.08	.57
PA advice red^n^	−0.32 (0.73)	−0.04	.66	−0.26 (0.81)		−0.03	.74	0.54 (0.88)		0.09	.54
Module choice diet^p^	−0.45 (0.31)	−0.11	.14	−0.26 (0.19)		−0.07	.18	−0.14 (0.51)		−0.02	.79
Module choice PA^p^	0.32 (0.54)	0.03	.55	—		—	—	0.29 (0.70)		0.03	.68
Module choice both^p^	−0.58 (0.27)	−0.17	.03^m^	—		—	—	—		—	—
Sessions diet	0.03 (0.07)	0.04	.61	−0.04 (0.10)		−0.02	.67	—		—	—
Sessions PA	0.15 (0.07)	0.15	.04^m^	—		—	—	0.27 (0.16)		0.13	.09

^a^The results’ interpretations are reported when all other predictors are held constant.

^b^Observations=291; *R*^2^/*R*^2^ adjusted=0.431/0.368; Akaike information criterion=1047.00.

^c^Observations=159; *R*^2^/*R*^2^ adjusted=0.669/0.607; Akaike information criterion=494.01.

^d^PA: physical activity.

^e^Observations=101; *R*^2^/*R*^2^ adjusted=0.726/0.630; Akaike information criterion=318.10.

^f^b: unstandardized regression coefficient.

^g^B: standardized regression coefficient.

^h^*Female* is the reference category.

^i^*Medium education* is the reference category.

^j^*Single* is the reference category.

^k^*Being unemployed* is the reference category.

^l^*No physical impairment* is the reference category.

^m^Values represent statistical significance.

^n^*Orange advice* is the reference category.

^o^These variables were not included in the model.

^p^*Choosing no module* is the reference category in the general intervention’s appreciation, whereas *choosing both modules* is the reference category for the appreciation of the diet module and the physical activity module.

## Discussion

### Principal Findings

This study has described the flow of participants in the MyLifestyleCoach intervention and identified characteristics related to this intervention’s use and appreciation. Our first aim was to describe the participants’ flow. Our findings resemble the typical nonusage attrition curve [3]. This was indicated by the largest drop in participation after the first session, and the attrition rate declined exponentially in subsequent sessions. More than half of the participants completed the entire 4 sessions in the case of 1 module or even 8 sessions in the case of both the diet and PA module intervention once they completed the first session of the module or modules. For instance, by implementing autonomy in our intervention and by offering participants a choice in which module or modules to participate, we expected to reduce this decline. Unfortunately, this was not the case, as only 20%-30% (diet module: 49/146, 33.6%; PA module: 2/12, 17%; both modules: 58/273, 21.2%) of participants completed their chosen module or modules. These numbers were (slightly) lower than those of other multisession PA interventions [34,35]. However, these interventions are not entirely comparable. Our intervention was more elaborate and did not only concern PA behavior but also concerned diet behavior. In addition, 2 findings regarding offering choices to the participants are worth mentioning; refer to the *Implications* section. When participants received a reminder email to revise their initial choice of following no module, it was remarkable that only 2 participants changed their initial module choice and decided to start a module. Another important finding was that more participants who indicated not to follow a module in the opening session were more likely to complete the follow-up questionnaire than participants who did not enter the opening session or did not make a module choice.

Our second aim was to examine which characteristics are linked to the use of initial and follow-up sessions. This study has demonstrated that age, marital status, health status, BMI, and physical impairment were related to key moments of stopping to use our intervention. In general, these findings are in line with previous literature [7,10,12,13,36]. Interestingly, a previous study by our research group discovered a trend that people with physical disabilities were less likely to choose the PA module on top of the diet module [22]. In this study, we found that they were more likely to complete the first session of the diet module when only the diet module was chosen. A reason for this could be that people with physical disabilities are more likely to focus and work on their dietary behavior, as they have fewer options to improve their PA because of their impairment. This finding shows the potential for specific groups in eHealth interventions in which people can choose the behavior or behaviors they prefer to work on. Furthermore, there seems to be a pattern that different motivation types are related to use at different points when people are likely to stop using the intervention. More autonomous motivation was associated with the completion of the opening session and the first session of the diet module. In contrast, participants who were more intrinsically motivated toward a healthy diet were less likely to complete these initial sessions. This finding is consistent with a study that found that users with higher levels of autonomous motivation toward dietary behaviors at baseline were less likely to stop using the intervention at an early moment [17]. Thus, perceiving eating healthily as a personally valued (and integrated) goal may be a relevant driver of initial use than the inherent joy of a healthy diet. On the other hand, more intrinsic motivation toward PA was associated with completing the opening session. This indicates that engaging in PA for inherent joy is relevant in initial use. Controlled motivation toward PA was linked to the starting and completion of both modules. These participants could have felt more pressure to start and complete the whole intervention by external regulations, for example, for a reward or introjected regulations for PA, for instance, to avoid negative feelings [37]. Thus, it is important to take the precise motivation type into account to stimulate use of the intervention.

Some program-related features are also linked to use, such as the advice a person received at the start of the intervention based on an assessment of the dietary and PA behavior on his or her initial behavioral performance. For instance, people with red advice for diet, indicating much room for improvement in their dietary behavior, were less likely to complete the whole intervention and thus both modules but were more likely to complete the diet module when only the diet module was chosen. The red diet advice seemed to increase participants’ focus to follow the diet sessions while decreasing their broader participation, possibly because of a high load or ego depletion and a lack of mental resources [38]. Therefore, it is important to keep in mind whether it is beneficial to provide advice in an intervention, as this may either have negative or positive effects on use. Another finding was that giving participants a choice when to start with the module was not predictive of completion. Thus, postponing the start of a module does not necessarily lead to nonusage attrition.

The third aim of this study was to describe the appreciation scores for the MyLifestyleCoach intervention and examine its predictors. First, the participants rated the intervention as reasonably positive. Second, no demographic factors were related to the appreciation scores. Regarding the motivational constructs, being less motivated toward being physically active, thus having higher amotivation, was associated with a higher appreciation of the diet module. These participants might be solely interested in the diet module and give a higher rating as a result. Having lower amotivation toward eating healthily was associated with a higher appreciation of the PA module. Those participants might value a healthy diet or even a healthy lifestyle, and as a result, provide a higher rating. Furthermore, more autonomous motivation toward being physically active was associated with a higher appreciation of the PA module. In addition, higher scores on basic psychological needs, particularly competence and relatedness, were linked to more favorable ratings of the program. Finally, some program features were also related to higher appreciation scores, such as the choice option. We found that choosing no module compared with both modules in the opening session and finishing more sessions in the PA module were related to a higher appreciation score. These evaluation scores, including an evaluation of the basic psychological needs of autonomy, competence, and relatedness, were assessed at the 6-month follow-up questionnaire. Here, a large proportion of participants (426/775, 55%) dropped out (see the *Limitations* section).

### Implications

Although our intervention structure may not necessarily be generalizable to other interventions, we provide some important implications that could be useful for the development of future interventions. First, we found that sending a generic reminder email to the participants whose initial choice was not to participate in the intervention minimally increased further intervention use, as only 2 participants revised their choice. Therefore, generic reminder emails are not recommended for this purpose. Instead, emails that contain new or different content or are tailored to specific characteristics, such as the extent of self-determination, might motivate people more to initiate a module [39,40].

Second, our results show that when people are given the option of beginning directly after the opening session or at a later moment, attrition rates are not negatively affected; however, this option does not improve use either. People possibly experience more autonomy by choosing the time point of using intervention parts, which might prevent them from stopping using the intervention and dropping out early. Thus, it can be assumed that providing participants with an option of when to start with the intervention is not detrimental for use.

Third, after people completed the first session of a module, about half of them finished the intervention comprising 4 or even 8 sessions spread over 3 months. It is recommended to make the first session of an intervention short and challenging and allow the person to choose small goals and achieve some success. However, more in-depth research is needed to examine why some individuals are more likely to adhere at particular moments within the intervention or give more favorable ratings. Further research should be undertaken to explore what could be improved to make participants more likely to adhere to the intervention. Examples of possible improvements could be more relevant content, better tailoring to specific groups, or using motivational interviewing to improve importance as early as possible.

Finally, the relative number of completed follow-up questionnaires was similar for the participants who chose to follow 1 or both modules and for those whose choice was to not follow any module. This latter group might have been more likely to stop using the intervention modules when this had been made obligatory and at risk of dropping out for the follow-up questionnaires. Thus, giving them a choice to start with the intervention, which is with a particular module, has prevented losing them to the follow-up questionnaires. This approach might have resulted in a slightly higher percentage of people who completed the follow-up questionnaire (349/775, 45% vs 409/987, 41.4%) compared with the previously tested single behavior *I Move* intervention [34].

### Limitations

There are several limitations worth mentioning. First, only self-reports were used to gather data. People could have responded in a more socially desirable way. For instance, they could have reported consuming more fruit and vegetables than they actually consume. This could have an effect on the received advice in the opening session [41]. Second, selection bias may have been present at some points of using attrition and dropout in this study. We cannot further investigate this as no information, such as demographics or motivation, is available for those who did not fill in the baseline questionnaire after randomization. It is likely that those who were not motivated to change their behavior more often dropped out. Third, this study focused on the theoretical framework of SDT, particularly focusing on motivation as a predictor of use and appreciation. Other psychosocial constructs, such as intention, could also be relevant to use. In a previous study, we found that these variables are highly correlated [22]. Therefore, we did not include these variables in our analyses to avoid multicollinearity. Finally, generalizability may be questioned, as a large part of our sample was highly educated. This is generally found in eHealth research (eg, the study by Rhodes et al [42]). Although our results demonstrate that education is not related to use at any point in this intervention, our predominantly highly educated sample could also have biased our findings. Nevertheless, future studies could aim to develop promotion strategies to attract more specific subgroups, such as less educated people with a less healthy lifestyle [43].

### Conclusions

This process evaluation study adds meaningful information on the use and appreciation of a web-based, computer-tailored dietary and PA intervention—*MyLifestyleCoach*. The results indicate that different types of motivation that were examined in this study at play at other moments where people are likely to stop using the intervention, such as the initial session or sessions or completing the whole intervention. Appreciation was associated with several motivational constructs, such as amotivation and intrinsic motivation, and related to basic psychological needs, such as competence. We derived some practical implications for developing eHealth interventions that contain multiple health behaviors. For instance, we found that about half of the participants ended the entire intervention once they finished the first session. Therefore, we recommend making the first session in a multiple-session intervention short, challenging, and rewarding and allow the person to choose small goals and achieve success.

## Appendix

Multimedia Appendix 1 Results from the stepwise logistic regression predicting completion of intervention components.

Multimedia Appendix 2 Results from the stepwise linear regression predicting the appreciation score for the whole intervention, the diet module, and physical activity module.

## Abbreviations

MVPA: moderate to vigorous physical activity
PA: physical activity
RCT: randomized controlled trial
SDT: self-determination theory

## References

[1] Kohl LF, Crutzen R, de Vries NK (2013). Online prevention aimed at lifestyle behaviors: a systematic review of reviews. J Med Internet Res.

[2] McVay MA, Bennett GG, Steinberg D, Voils CI (2019). Dose-response research in digital health interventions: concepts, considerations, and challenges. Health Psychol.

[3] Eysenbach G (2005). The law of attrition. J Med Internet Res.

[4] Kelders SM, Kok RN, Ossebaard HC, Van Gemert-Pijnen JE (2012). Persuasive system design does matter: a systematic review of adherence to web-based interventions. J Med Internet Res.

[5] Glasgow RE, Emmons KM (2007). How can we increase translation of research into practice? Types of evidence needed. Annu Rev Public Health.

[6] Robroek SJ, Brouwer W, Lindeboom D, Oenema A, Burdorf A (2010). Demographic, behavioral, and psychosocial correlates of using the website component of a worksite physical activity and healthy nutrition promotion program: a longitudinal study. J Med Internet Res.

[7] Schneider F, van Osch L, Schulz DN, Kremers SP, de Vries H (2012). The influence of user characteristics and a periodic email prompt on exposure to an internet-delivered computer-tailored lifestyle program. J Med Internet Res.

[8] Schulz DN, Schneider F, de Vries H, van Osch LA, van Nierop PW, Kremers SP (2012). Program completion of a web-based tailored lifestyle intervention for adults: differences between a sequential and a simultaneous approach. J Med Internet Res.

[9] Strecher VJ, McClure J, Alexander G, Chakraborty B, Nair V, Konkel J, Greene S, Couper M, Carlier C, Wiese C, Little R, Pomerleau C, Pomerleau O (2008). The role of engagement in a tailored web-based smoking cessation program: randomized controlled trial. J Med Internet Res.

[10] Van't Riet J, Crutzen R, De Vries H (2010). Investigating predictors of visiting, using, and revisiting an online health-communication program: a longitudinal study. J Med Internet Res.

[11] Van der Mispel C, Poppe L, Crombez G, Verloigne M, De Bourdeaudhuij I (2017). A self-regulation-based eHealth intervention to promote a healthy lifestyle: investigating user and website characteristics related to attrition. J Med Internet Res.

[12] Brouwer W, Oenema A, Raat H, Crutzen R, de Nooijer J, de Vries NK, Brug J (2010). Characteristics of visitors and revisitors to an internet-delivered computer-tailored lifestyle intervention implemented for use by the general public. Health Educ Res.

[13] Reinwand DA, Schulz DN, Crutzen R, Kremers SP, de Vries H (2015). Who follows eHealth interventions as recommended? A study of participants' personal characteristics from the experimental arm of a randomized controlled trial. J Med Internet Res.

[14] Leung AW, Chan RS, Sea MM, Woo J (2017). An overview of factors associated with adherence to lifestyle modification programs for weight management in adults. Int J Environ Res Public Health.

[15] Rothman A, Baldwin A, Hertel A, Fuglestad P (2011). Self-regulation and behavior change: disentangling behavioral initiation and behavioral maintenance. Handbook of Self-Regulation: Research, Theory, and Applications.

[16] Kwasnicka D, Dombrowski SU, White M, Sniehotta F (2016). Theoretical explanations for maintenance of behaviour change: a systematic review of behaviour theories. Health Psychol Rev.

[17] Coa K, Patrick H (2016). Baseline motivation type as a predictor of dropout in a healthy eating text messaging program. JMIR Mhealth Uhealth.

[18] Ryan RM, Patrick H, Deci EL, Williams GC (2008). Facilitating health behaviour change and its maintenance: interventions based on self-determination theory. Eur Health Psychol.

[19] Fortier MS, Duda JL, Guerin E, Teixeira PJ (2012). Promoting physical activity: development and testing of self-determination theory-based interventions. Int J Behav Nutr Phys Act.

[20] Ryan RM, Deci EL (2000). Self-determination theory and the facilitation of intrinsic motivation, social development, and well-being. Am Psychol.

[21] Deci EL, Ryan RM (2008). Self-determination theory: a macrotheory of human motivation, development, and health. Can Psychol.

[22] Coumans JM, Bolman CA, Oenema A, Lechner L (2020). Predictors of self-determined module choice in a web-based computer-tailored diet and physical activity intervention: secondary analysis of data from a randomized controlled trial. J Med Internet Res.

[23] Friederichs SA, Oenema A, Bolman C, Guyaux J, van Keulen HM, Lechner L (2014). I Move: systematic development of a web-based computer tailored physical activity intervention, based on motivational interviewing and self-determination theory. BMC Public Health.

[24] Coumans JM, Bolman CA, Friederichs SA, Oenema A, Lechner L (2020). Development and testing of a personalized web-based diet and physical activity intervention based on motivational interviewing and the self-determination theory: protocol for the MyLifestyleCoach randomized controlled trial. JMIR Res Protoc.

[25] Bartholomew LK, Markham CM, Ruiter RA, Fernàndez ME, Kok G, Parcel GS (2016). Planning Health Promotion Programs: An Intervention Mapping Approach. Fourth edition.

[26] Brink E, van Rossum C, Postma-Smeets A, Stafleu A, Wolvers D, van Dooren C, Toxopeus I, Buurma-Rethans E, Geurts M, Ocké M (2019). Development of healthy and sustainable food-based dietary guidelines for the Netherlands. Public Health Nutr.

[27] Weggemans RM, Backx FJ, Borghouts L, Chinapaw M, Hopman MT, Koster A, Kremers S, van Loon LJ, May A, Mosterd A, van der Ploeg HP, Takken T, Visser M, Wendel-Vos GC, de Geus EJ, Committee Dutch Physical Activity Guidelines 2017 (2018). The 2017 Dutch physical activity guidelines. Int J Behav Nutr Phys Act.

[28] Levesque CS, Williams GC, Elliot D, Pickering MA, Bodenhamer B, Finley PJ (2007). Validating the theoretical structure of the Treatment Self-Regulation Questionnaire (TSRQ) across three different health behaviors. Health Educ Res.

[29] Markland D, Tobin V (2004). A modification to the behavioural regulation in exercise questionnaire to include an assessment of amotivation. J Sport Exerc Psychol.

[30] Huybrechts I, Börnhorst C, Pala V, Moreno LA, Barba G, Lissner L, Fraterman A, Veidebaum T, Hebestreit A, Sieri S, Ottevaere C, Tornaritis M, Molnár D, Ahrens W, De Henauw S, IDEFICS Consortium (2011). Evaluation of the Children's Eating Habits Questionnaire used in the IDEFICS study by relating urinary calcium and potassium to milk consumption frequencies among European children. Int J Obes (Lond).

[31] Coumans JM, Danner UN, Intemann T, De Decker A, Hadjigeorgiou C, Hunsberger M, Moreno LA, Russo P, Stomfai S, Veidebaum T, Adan RA, Hebestreit A, I.Family Consortium (2018). Emotion-driven impulsiveness and snack food consumption of European adolescents: results from the I.Family study. Appetite.

[32] Wendel-Vos GC, Schuit AJ, Saris WH, Kromhout D (2003). Reproducibility and relative validity of the short questionnaire to assess health-enhancing physical activity. J Clin Epidemiol.

[33] Walthouwer MJ, Oenema A, Lechner L, de Vries H (2015). Comparing a video and text version of a web-based computer-tailored intervention for obesity prevention: a randomized controlled trial. J Med Internet Res.

[34] Friederichs SA, Oenema A, Bolman C, Lechner L (2015). Long term effects of self-determination theory and motivational interviewing in a web-based physical activity intervention: randomized controlled trial. Int J Behav Nutr Phys Act.

[35] Edney S, Ryan JC, Olds T, Monroe C, Fraysse F, Vandelanotte C, Plotnikoff R, Curtis R, Maher C (2019). User engagement and attrition in an app-based physical activity intervention: secondary analysis of a randomized controlled trial. J Med Internet Res.

[36] Ahnis A, Riedl A, Figura A, Steinhagen-Thiessen E, Liebl ME, Klapp BF (2012). Psychological and sociodemographic predictors of premature discontinuation of a 1-year multimodal outpatient weight-reduction program: an attrition analysis. Patient Prefer Adherence.

[37] Vallerand R (1997). Toward a hierarchical model of intrinsic and extrinsic motivation. Adv Exp Soc Psychol.

[38] Baumeister RF, Bratslavsky E, Muraven M, Tice DM (1998). Ego depletion: is the active self a limited resource?. J Pers Soc Psychol.

[39] Fry JP, Neff RA (2009). Periodic prompts and reminders in health promotion and health behavior interventions: systematic review. J Med Internet Res.

[40] Woodall WG, Buller DB, Saba L, Zimmerman D, Waters E, Hines JM, Cutter GR, Starling R (2007). Effect of emailed messages on return use of a nutrition education website and subsequent changes in dietary behavior. J Med Internet Res.

[41] Hebert JR, Hurley TG, Peterson KE, Resnicow K, Thompson FE, Yaroch AL, Ehlers M, Midthune D, Williams GC, Greene GW, Nebeling L (2008). Social desirability trait influences on self-reported dietary measures among diverse participants in a multicenter multiple risk factor trial. J Nutr.

[42] Rhodes SD, Bowie DA, Hergenrather KC (2003). Collecting behavioural data using the world wide web: considerations for researchers. J Epidemiol Community Health.

[43] Pampel FC, Krueger PM, Denney JT (2010). Socioeconomic disparities in health behaviors. Annu Rev Sociol.

