# Supplementary material for: Use and Appreciation of a Web-Based, Computer-Tailored Diet and Physical Activity Intervention Based on the Self-determination Theory: Evaluation Study of Process and Predictors
Source: JMIR Form Res. 2021 Dec 2;5(12):e22390. doi: 10.2196/22390 (PMC8686464; doi:10.2196/22390)
Supplement: Multimedia Appendix 1 [file formative_v5i12e22390_app1.docx]

Multimedia Appendix 1. Results step-wise logistic regression predicting completion of intervention components.

**Table 1.** Results step-wise logistic regression predicting completion of the opening session (N = 775)

| Predictors | Completed opening session  Model 1 | | | Completed opening session Model 2 | | |
| --- | --- | --- | --- | --- | --- | --- |
|  | OR (95% CI) | SE | *P* value | OR (95% CI) | SE | *P* value |
| Intercept | 1.12 (0.25-4.94) | 0.76 | 0.882 | 1.31 (0.21-8.09) | 0.93 | 0.772 |
| Age | 0.998 (0.98-1.01) | 0.01 | 0.814 | 1.003 (0.99-1.02) | 0.01 | 0.691 |
| Gender^a^ | 1.39 (0.97-1.98) | 0.18 | 0.069 | 1.27 (0.88-1.84) | 0.19 | 0.202 |
| Education high^b^ | 0.99 (0.67-1.46) | 0.20 | 0.957 | 0.98 (0.66-1.47) | 0.21 | 0.931 |
| Education low^b^ | 0.90 (0.36-2.27) | 0.47 | 0.820 | 1.09 (0.4-2.94) | 0.51 | 0.864 |
| Marital status partner^c^ | 0.61 (0.42-0.91) | 0.20 | **0.014** | 0.60 (0.41-0.89) | 0.20 | **0.012** |
| Work employed^d^ | 1.01 (0.70-1.45) | 0.18 | 0.961 | 0.97 (0.67-1.41) | 0.19 | 0.874 |
| Impairment^e^ | 1.59 (0.62-4.09) | 0.48 | 0.337 | 1.74 (0.66-4.57) | 0.49 | 0.259 |
| BMI | 1.04 (1.01-1.08) | 0.02 | **0.024** | 1.04 (0.996-1.08) | 0.02 | 0.079 |
| Health status | 1.002 (0.99-1.01) | 0.01 | 0.764 | 1.0005 (0.99-1.01) | 0.01 | 0.941 |
|  |  |  |  |  |  |  |
| Amotivation Diet |  |  |  | 1.09 (0.88-1.33) | 0.10 | 0.431 |
| Amotivation PA |  |  |  | 0.90 (0.74-1.1) | 0.10 | 0.300 |
| Controlled Motivation Diet |  |  |  | 0.95 (0.75-1.2) | 0.12 | 0.678 |
| Controlled Motivation PA |  |  |  | 1.12 (0.89-1.42) | 0.12 | 0.329 |
| Autonomous Motivation Diet |  |  |  | 1.35 (1.05-1.74) | 0.13 | **0.020** |
| Autonomous Motivation PA |  |  |  | 0.80 (0.61-1.04) | 0.13 | 0.098 |
| Intrinsic Motivation Diet |  |  |  | 0.60 (0.48-0.76) | 0.12 | **<0.001** |
| Intrinsic Motivation PA |  |  |  | 1.38 (1.13-1.68) | 0.10 | **0.001** |
| R^2^ Tjur | 0.019 | | | 0.054 | | |
| AIC | 881.122 | | | 869.586 | | |

*Note*. Values in bold represent significance. Results’ interpretations are reported when all other predictors were held constant. When cells in the table are blank, these variables were not included in the model. OR = odds ratio; SE = standard error; R^2^ = explained variance; AIC = Akaike information criterion.

^a^ Female is the reference category

^b^ Medium education is the reference category

^c^ Single is the reference category

^d^ Being unemployed is the reference category

^e^ No physical impairment is the reference category

**Table 2.** Results step-wise logistic regression predicting completing the first session of the diet module (*n* = 146)

| Predictors | Completed session 1 | | | Completed session 1 | | | Completed session 1 | | |
| --- | --- | --- | --- | --- | --- | --- | --- | --- | --- |
|  | OR (95% CI) | SE | *P* value | OR (95% CI) | SE | *P*  value | OR (95% CI) | SE | *P*  value |
| Intercept | 5.02 (0.27-93.75) | 1.49 | 0.280 | 10.87  (0.19-613.74) | 2.06 | 0.246 | 2.88  (0.04-221.08) | 2.22 | 0.633 |
| Age | 1.01 (0.98-1.04) | 0.01 | 0.494 | 1.01 (0.98-1.04) | 0.02 | 0.642 | 1.02 (0.99-1.06) | 0.02 | 0.264 |
| Gender^a^ | 0.86 (0.42-1.76) | 0.36 | 0.683 | 0.74 (0.33-1.63) | 0.40 | 0.448 | 0.69 (0.31-1.56) | 0.41 | 0.378 |
| Education high^b^ | 0.67 (0.28-1.58) | 0.44 | 0.357 | 0.78 (0.31-1.99) | 0.47 | 0.609 | 1.02 (0.38-2.73) | 0.50 | 0.963 |
| Education low^b^ | 1.28 (0.18-9.27) | 1.01 | 0.805 | 1.58 (0.19-13.11) | 1.08 | 0.673 | 1.53 (0.19-12.44) | 1.07 | 0.692 |
| Marital status partner^c^ | 1.14 (0.52-2.51) | 0.40 | 0.739 | 0.96 (0.42-2.22) | 0.43 | 0.926 | 1.03 (0.43-2.45) | 0.44 | 0.947 |
| Work employed^d^ | 1.68 (0.76-3.71) | 0.41 | 0.203 | 1.38 (0.6-3.21) | 0.43 | 0.451 | 1.26 (0.53-3.01) | 0.44 | 0.601 |
| Impairment^e^ | 4.79 (0.74-30.76) | 0.95 | 0.099 | 6.54 (0.86-49.96) | 1.04 | 0.070 | 9.05 (1.06-77.1) | 1.09 | **0.044** |
| BMI | 0.92 (0.85-1.003) | 0.04 | 0.059 | 0.89 (0.81-0.98) | 0.05 | **0.015** | 0.87 (0.79-0.97) | 0.05 | **0.009** |
| Health status | 1.002 (0.98-1.03) | 0.01 | 0.905 | 1.01 (0.98-1.05) | 0.02 | 0.387 | 1.02 (0.99-1.05) | 0.02 | 0.253 |
|  |  |  |  |  |  |  |  |  |  |
| Amotivation Diet |  |  |  | 0.83 (0.50-1.36) | 0.26 | 0.454 | 0.83 (0.49-1.39) | 0.26 | 0.469 |
| Amotivation PA |  |  |  | 1.37 (0.88-2.14) | 0.23 | 0.164 | 1.50 (0.95-2.37) | 0.23 | 0.084 |
| Controlled Motivation Diet |  |  |  | 1.18 (0.67-2.09) | 0.29 | 0.570 | 1.24 (0.68-2.25) | 0.30 | 0.477 |
| Controlled Motivation PA |  |  |  | 0.70 (0.41-1.22) | 0.28 | 0.209 | 0.65 (0.37-1.15) | 0.29 | 0.139 |
| Autonomous Motivation Diet |  |  |  | 2.04 (1.08-3.86) | 0.32 | **0.028** | 2.27 (1.17-4.41) | 0.34 | **0.016** |
| Autonomous Motivation PA |  |  |  | 0.73 (0.39-1.36) | 0.32 | 0.322 | 0.69 (0.36-1.33) | 0.33 | 0.271 |
| Intrinsic Motivation Diet |  |  |  | 0.56 (0.33-0.95) | 0.27 | **0.030** | 0.53 (0.31-0.91) | 0.27 | **0.020** |
| Intrinsic Motivation PA |  |  |  | 0.94 (0.59-1.49) | 0.24 | 0.781 | 1.05 (0.65-1.72) | 0.25 | 0.832 |
|  |  |  |  |  |  |  |  |  |  |
| Diet guideline red^f^ |  |  |  |  |  |  | 2.77 (0.98-7.86) | 0.53 | 0.055 |
| Start later^g^ |  |  |  |  |  |  | 0.55 (0.25-1.22) | 0.40 | 0.143 |
| R^2^ Tjur | 0.045 | | | 0.124 | | | 0.158 | | |
| AIC | 212.32 | | | 216.34 | | | 215.19 | | |

*Note*. Values in bold represent significance. Results’ interpretations are reported when all other predictors were held constant. When cells in the table are blank, these variables were not included in the model. OR = odds ratio; SE = standard error; R^2^ = explained variance; AIC = Akaike information criterion.

^a^ Female is the reference category

^b^ Medium education is the reference category

^c^ Single is the reference category

^d^ Being unemployed is the reference category

^e^ No physical impairment is the reference category

^f^ Orange advice is the reference category

^g^ Directly starting with the first session was the reference category

**Table 3.** Results step-wise logistic regression predicting completing the first session of both modules (*n* = 273)

| Predictors | Completed sessions 1 | | | Completed sessions 1 | | | Completed sessions 1 | | |
| --- | --- | --- | --- | --- | --- | --- | --- | --- | --- |
|  | OR (95% CI) | SE | *P* value | OR (95% CI) | SE | *P*  value | OR (95% CI) | SE | *P*  value |
| Intercept | 0.12 (0.01-1.06) | 1.09 | 0.056 | 0.29 (0.02-4.47) | 1.39 | 0.379 | 0.06 (0.002-2.39) | 1.85 | 0.136 |
| Age | 1.01 (0.99-1.03) | 0.01 | 0.405 | 1.02 (0.99-1.04) | 0.01 | 0.138 | 1.02 (0.99-1.04) | 0.01 | 0.145 |
| Gender^a^ | 0.76 (0.43-1.33) | 0.29 | 0.336 | 0.68 (0.37-1.23) | 0.30 | 0.200 | 0.70 (0.38-1.31) | 0.32 | 0.266 |
| Education high^b^ | 1.34 (0.75-2.38) | 0.29 | 0.322 | 1.22 (0.66-2.27) | 0.32 | 0.527 | 1.39 (0.73-2.66) | 0.33 | 0.320 |
| Education low^b^ | 0.94 (0.21-4.19) | 0.76 | 0.939 | 1.28 (0.28-5.94) | 0.78 | 0.749 | 1.21 (0.25-5.91) | 0.81 | 0.817 |
| Marital status partner^c^ | 0.52 (0.30-0.91) | 0.29 | **0.022** | 0.55 (0.31-0.98) | 0.29 | **0.042** | 0.52 (0.28-0.95) | 0.31 | **0.034** |
| Work employed^d^ | 0.78 (0.44-1.35) | 0.28 | 0.370 | 0.73 (0.41-1.30) | 0.30 | 0.280 | 0.84 (0.46-1.54) | 0.31 | 0.574 |
| Impairment^e^ | 3.44 (1.06-11.17) | 0.60 | **0.040** | 2.54  (0.75-8.59) | 0.62 | 0.135 | 3.04 (0.79-11.75) | 0.69 | 0.106 |
| BMI | 0.99 (0.95-1.04) | 0.02 | 0.816 | 0.99 (0.94-1.04) | 0.03 | 0.680 | 0.98 (0.93-1.04) | 0.03 | 0.553 |
| Health status | 1.03 (1.01-1.05) | 0.01 | **0.005** | 1.03 (1.01-1.05) | 0.01 | **0.005** | 1.04 (1.01-1.06) | 0.01 | **0.001** |
|  |  |  |  |  |  |  |  |  |  |
| Amotivation Diet |  |  |  | 1.24 (0.90-1.71) | 0.16 | 0.195 | 1.19 (0.84-1.67) | 0.17 | 0.329 |
| Amotivation PA |  |  |  | 0.67 (0.47-0.96) | 0.18 | **0.030** | 0.72 (0.49-1.06) | 0.20 | 0.096 |
| Controlled Motivation Diet |  |  |  | 0.70 (0.48-1.03) | 0.20 | 0.074 | 0.70 (0.47-1.06) | 0.21 | 0.092 |
| Controlled Motivation PA |  |  |  | 1.59 (1.08-2.33) | 0.20 | **0.019** | 1.70 (1.13-2.57) | 0.21 | **0.011** |
| Autonomous Motivation Diet |  |  |  | 0.96 (0.63-1.48) | 0.22 | 0.854 | 0.995 (0.64-1.55) | 0.23 | 0.983 |
| Autonomous Motivation PA |  |  |  | 0.97 (0.62-1.53) | 0.23 | 0.901 | 0.97 (0.61-1.55) | 0.24 | 0.900 |
| Intrinsic Motivation Diet |  |  |  | 1.02 (0.72-1.44) | 0.18 | 0.914 | 0.94 (0.66-1.33) | 0.18 | 0.714 |
| Intrinsic Motivation PA |  |  |  | 0.81 (0.59-1.10) | 0.16 | 0.170 | 0.80 (0.58-1.11) | 0.17 | 0.189 |
|  |  |  |  |  |  |  |  |  |  |
| Diet guideline green^f,g^ |  |  |  |  |  |  | 0.00 (0-Inf) | 974.90 | 0.987 |
| Diet guideline red^f^ |  |  |  |  |  |  | 0.59 (0.31-1.12) | 0.33 | 0.109 |
| PA guideline green^f^ |  |  |  |  |  |  | 3.18 (0.4-24.92) | 1.05 | 0.271 |
| PA guideline red^f^ |  |  |  |  |  |  | 16.82 (1.28-221.17) | 1.31 | **0.032** |
| First module - PA^h^ |  |  |  |  |  |  | 0.21 (0.07-0.63) | 0.56 | **0.005** |
| R^2^ Tjur | 0.072 | | | 0.108 | | | 0.167 | | |
| AIC | 370.75 | | | 375.58 | | | 366.99 | | |

*Note*. Values in bold represent significance. Results’ interpretations are reported when all other predictors were held constant. When cells in the table are blank, these variables were not included in the model. OR = odds ratio; SE = standard error; IM = intrinsic motivation; R^2^ = explained variance; AIC = Akaike information criterion.

^a^ Female is the reference category

^b^ Medium education is the reference category

^c^ Single is the reference category

^d^ Being unemployed is the reference category

^e^ No physical impairment is the reference category

^f^ Orange advice is the reference category

^g^ Only two participants received green advice. Consequently, the OR and SE are less reliable.

^h^ Choosing the diet module to start with when both modules were chosen was the reference category

**Table 4.** Results step-wise logistic regression predicting completing all session (yes/no) of the diet module (*n* = 146)

| Predictors | Completed all sessions | | | Completed all sessions | | | Completed all sessions | | |
| --- | --- | --- | --- | --- | --- | --- | --- | --- | --- |
|  | OR (95% CI) | SE | *P* value | OR (95% CI) | SE | *P*  value | OR (95% CI) | SE | *P*  value |
| Intercept | 0.29 (0.01-6.32) | 1.58 | 0.430 | 0.28 (0.01-14.46) | 2.02 | 0.525 | 0.05 (0.001-3.91) | 2.25 | 0.176 |
| Age | 1.03 (0.995-1.06) | 0.02 | 0.098 | 1.02 (0.99-1.06) | 0.02 | 0.216 | 1.04 (0.998-1.08) | 0.02 | 0.065 |
| Gender^a^ | 0.95 (0.45-1.98) | 0.38 | 0.890 | 1.01 (0.45-2.25) | 0.41 | 0.980 | 0.99 (0.44-2.22) | 0.41 | 0.977 |
| Education high^b^ | 0.99 (0.42-2.38) | 0.44 | 0.991 | 1.16 (0.47-2.88) | 0.46 | 0.746 | 1.55 (0.6-4.04) | 0.49 | 0.366 |
| Education low^b^ | 1.37 (0.19-9.95) | 1.01 | 0.754 | 1.34 (0.17-10.66) | 1.06 | 0.779 | 1.32 (0.17-10.57) | 1.06 | 0.791 |
| Marital status partner^c^ | 0.92 (0.41-2.07) | 0.41 | 0.834 | 0.83 (0.36-1.91) | 0.43 | 0.663 | 0.91 (0.38-2.18) | 0.44 | 0.840 |
| Work employed^d^ | 1.16 (0.51-2.64) | 0.42 | 0.721 | 0.97 (0.41-2.29) | 0.44 | 0.951 | 0.85 (0.35-2.07) | 0.45 | 0.722 |
| Impairment^e^ | 0.94 (0.19-4.61) | 0.81 | 0.938 | 0.82 (0.15-4.47) | 0.87 | 0.818 | 0.88 (0.14-5.32) | 0.92 | 0.885 |
| BMI | 0.97 (0.89-1.06) | 0.04 | 0.526 | 0.96 (0.88-1.05) | 0.05 | 0.407 | 0.95 (0.86-1.05) | 0.05 | 0.322 |
| Health status | 0.999 (0.97-1.03) | 0.01 | 0.939 | 1.003 (0.97-1.03) | 0.01 | 0.840 | 1.01 (0.98-1.04) | 0.02 | 0.635 |
|  |  |  |  |  |  |  |  |  |  |
| Amotivation Diet |  |  |  | 1.21 (0.73-2.00) | 0.26 | 0.458 | 1.19 (0.71-1.99) | 0.26 | 0.513 |
| Amotivation PA |  |  |  | 1.07 (0.70-1.64) | 0.22 | 0.757 | 1.19 (0.76-1.85) | 0.23 | 0.450 |
| Controlled Motivation Diet |  |  |  | 0.99 (0.56-1.73) | 0.29 | 0.960 | 1.09 (0.60-1.97) | 0.30 | 0.786 |
| Controlled Motivation PA |  |  |  | 0.71 (0.41-1.23) | 0.28 | 0.220 | 0.63 (0.35-1.13) | 0.30 | 0.121 |
| Autonomous Motivation Diet |  |  |  | 1.39 (0.76-2.57) | 0.31 | 0.286 | 1.55 (0.82-2.95) | 0.33 | 0.179 |
| Autonomous Motivation PA |  |  |  | 0.86 (0.47-1.57) | 0.31 | 0.627 | 0.82 (0.44-1.53) | 0.32 | 0.524 |
| Intrinsic Motivation Diet |  |  |  | 0.995 (0.59-1.67) | 0.27 | 0.986 | 0.96 (0.56-1.64) | 0.28 | 0.878 |
| Intrinsic Motivation PA |  |  |  | 0.93 (0.59-1.49) | 0.24 | 0.776 | 1.07 (0.65-1.75) | 0.25 | 0.791 |
|  |  |  |  |  |  |  |  |  |  |
| Diet guideline red^f^ |  |  |  |  |  |  | 3.17 (1.11-9.04) | 0.53 | **0.031** |
| Start later^g^ |  |  |  |  |  |  | 0.56 (0.26-1.23) | 0.40 | 0.148 |
| R^2^ Tjur | 0.025 | | | 0.061 | | | 0.099 | | |
| AIC | 202.76 | | | 213.35 | | | 211.14 | | |

*Note*. Values in bold represent significance. Results’ interpretations are reported when all other predictors were held constant. When cells in the table are blank, these variables were not included in the model. OR = odds ratio; SE = standard error; R^2^ = explained variance; AIC = Akaike information criterion.

^a^ Female is the reference category

^b^ Medium education is the reference category

^c^ Single is the reference category

^d^ Being unemployed is the reference category

^e^ No physical impairment is the reference category

^f^ Orange advice is the reference category

^g^ Directly starting with the first session was the reference category

**Table 5.** Results step-wise logistic regression predicting completing all session (yes/no) of both modules (*n* = 273)

| Predictors | Completed all sessions | | | Completed all sessions | | | Completed all sessions | | |
| --- | --- | --- | --- | --- | --- | --- | --- | --- | --- |
|  | OR (95% CI) | SE | *P* value | OR (95% CI) | SE | *P*  value | OR (95% CI) | SE | *P*  value |
| Intercept | 0.15 (0.01-1.88) | 1.28 | 0.143 | 0.35 (0.02-8.30) | 1.61 | 0.518 | 0.22 (0.003-14.94) | 2.16 | 0.478 |
| Age | 1.01 (0.99-1.04) | 0.01 | 0.270 | 1.03 (1.004-1.07) | 0.02 | **0.026** | 1.03 (1.002-1.07) | 0.02 | **0.035** |
| Gender^a^ | 1.30 (0.68-2.48) | 0.33 | 0.432 | 1.01 (0.5-2.03) | 0.35 | 0.977 | 1.08 (0.52-2.25) | 0.37 | 0.831 |
| Education high^b^ | 1.03 (0.52-2.05) | 0.35 | 0.932 | 1.22 (0.57-2.6) | 0.39 | 0.607 | 1.34 (0.61-2.96) | 0.40 | 0.466 |
| Education low^b^ | 0.91 (0.17-5.06) | 0.87 | 0.919 | 1.36 (0.21-8.93) | 0.96 | 0.748 | 1.12 (0.15-8.1) | 1.01 | 0.911 |
| Marital status partner^c^ | 0.55 (0.28-1.05) | 0.33 | 0.069 | 0.59 (0.30-1.17) | 0.35 | 0.132 | 0.51 (0.25-1.04) | 0.37 | 0.063 |
| Work employed^d^ | 0.67 (0.35-1.29) | 0.33 | 0.235 | 0.62 (0.31-1.24) | 0.35 | 0.176 | 0.73 (0.36-1.49) | 0.36 | 0.391 |
| Impairment^e^ | 1.72 (0.50-5.91) | 0.63 | 0.392 | 1.31 (0.35-4.96) | 0.68 | 0.688 | 1.82 (0.36-9.13) | 0.82 | 0.465 |
| BMI | 0.97 (0.92-1.03) | 0.03 | 0.379 | 0.96 (0.90-1.02) | 0.03 | 0.222 | 0.95 (0.89-1.02) | 0.03 | 0.144 |
| Health status | 1.01 (0.99-1.04) | 0.01 | 0.209 | 1.01 (0.99-1.04) | 0.01 | 0.276 | 1.02 (0.99-1.04) | 0.01 | 0.170 |
|  |  |  |  |  |  |  |  |  |  |
| Amotivation Diet |  |  |  | 1.36 (0.93-1.99) | 0.19 | 0.108 | 1.27 (0.85-1.89) | 0.20 | 0.248 |
| Amotivation PA |  |  |  | 0.79 (0.53-1.19) | 0.21 | 0.263 | 0.95 (0.60-1.49) | 0.23 | 0.815 |
| Controlled Motivation Diet |  |  |  | 0.48 (0.29-0.79) | 0.26 | **0.004** | 0.43 (0.25-0.75) | 0.28 | **0.003** |
| Controlled Motivation PA |  |  |  | 2.27 (1.36-3.79) | 0.26 | **0.002** | 2.60 (1.48-4.59) | 0.29 | **0.001** |
| Autonomous Motivation Diet |  |  |  | 0.92 (0.57-1.5) | 0.25 | 0.752 | 0.92 (0.56-1.51) | 0.25 | 0.742 |
| Autonomous Motivation PA |  |  |  | 0.86 (0.51-1.46) | 0.27 | 0.580 | 0.89 (0.52-1.53) | 0.28 | 0.672 |
| Intrinsic Motivation Diet |  |  |  | 0.92 (0.60-1.39) | 0.21 | 0.685 | 0.83 (0.53-1.30) | 0.23 | 0.416 |
| Intrinsic Motivation PA |  |  |  | 0.91 (0.63-1.33) | 0.19 | 0.632 | 0.92 (0.62-1.39) | 0.21 | 0.704 |
|  |  |  |  |  |  |  |  |  |  |
| Diet guideline green^f^ |  |  |  |  |  |  | 0.00 (0-Inf) | 951.69 | 0.988 |
| Diet guideline red^f^ |  |  |  |  |  |  | 0.39 (0.16-0.9) | 0.43 | **0.028** |
| PA guideline green^f^ |  |  |  |  |  |  | 1.69 (0.15-19.4) | 1.24 | 0.673 |
| PA guideline red^f^ |  |  |  |  |  |  | 9.89 (0.50-194.1) | 1.52 | 0.131 |
| First module - PA^g^ |  |  |  |  |  |  | 0.05 (0.01-0.5) | 1.16 | **0.010** |
| R^2^ Tjur | 0.045 | | | 0.118 | | | 0.188 | | |
| AIC | 289.65 | | | 287.71 | | | 276.69 | | |

*Note*. Values in bold represent significance. Results’ interpretations are reported when all other predictors were held constant. When cells in the table are blank, these variables were not included in the model. OR = odds ratio; SE = standard error; R^2^ = explained variance; AIC = Akaike information criterion.

^a^ Female is the reference category

^b^ Medium education is the reference category

^c^ Single is the reference category

^d^ Being unemployed is the reference category

^e^ No physical impairment is the reference category

^f^ Orange advice is the reference category

^g^ Choosing the diet module to start with when both modules were chosen was the reference category
