# Supplementary material for: Use and Appreciation of a Web-Based, Computer-Tailored Diet and Physical Activity Intervention Based on the Self-determination Theory: Evaluation Study of Process and Predictors
Source: JMIR Form Res. 2021 Dec 2;5(12):e22390. doi: 10.2196/22390 (PMC8686464; doi:10.2196/22390)
Supplement: Multimedia Appendix 2 [file formative_v5i12e22390_app2.docx]

Multimedia Appendix 2. Results from the stepwise linear regression predicting the appreciation score for the whole intervention, the diet module, and PA module.

**Table 1.** Results from the stepwise linear regression predicting the general intervention’s appreciation score

|  | Appreciation | | | | Appreciation | | | | Appreciation | | | |
| --- | --- | --- | --- | --- | --- | --- | --- | --- | --- | --- | --- | --- |
| *Predictors* | b | SE | B | *P* value | b | SE | B | *P* value | b | SE | B | *P* value |
| Intercept | 4.99 | 0.90 | 0.00 | **<0.001** | 0.46 | 0.99 | 0.00 | 0.645 | 0.43 | 1.17 | 0.00 | 0.712 |
| Age | 0.01 | 0.01 | 0.11 | 0.110 | 0.01 | 0.01 | 0.06 | 0.260 | 0.01 | 0.01 | 0.06 | 0.347 |
| Gender^a^ | -0.17 | 0.22 | -0.05 | 0.437 | -0.08 | 0.18 | -0.02 | 0.659 | -0.11 | 0.18 | -0.03 | 0.561 |
| Education high^b^ | -0.14 | 0.24 | -0.04 | 0.572 | 0.11 | 0.21 | 0.03 | 0.597 | 0.14 | 0.21 | 0.04 | 0.501 |
| Education low^b^ | 0.75 | 0.53 | 0.09 | 0.161 | 0.24 | 0.45 | 0.03 | 0.595 | 0.28 | 0.46 | 0.03 | 0.542 |
| Marital partner^c^ | 0.34 | 0.23 | 0.09 | 0.136 | 0.16 | 0.19 | 0.04 | 0.398 | 0.21 | 0.19 | 0.06 | 0.286 |
| Work^d^ | 0.02 | 0.22 | 0.005 | 0.941 | -0.03 | 0.19 | -0.01 | 0.876 | 0.0003 | 0.19 | 0.0001 | 0.999 |
| Impairment^e^ | -0.27 | 0.48 | -0.04 | 0.568 | -0.71 | 0.40 | -0.09 | 0.077 | -0.73 | 0.41 | -0.10 | 0.075 |
| BMI | 0.01 | 0.02 | 0.02 | 0.746 | 0.01 | 0.02 | 0.02 | 0.696 | 0.01 | 0.02 | 0.03 | 0.550 |
| Health status | 0.01 | 0.01 | 0.11 | 0.096 | 0.01 | 0.01 | 0.08 | 0.158 | 0.006 | 0.01 | 0.04 | 0.518 |
|  |  |  |  |  |  |  |  |  |  |  |  |  |
| Amotivation Diet |  |  |  |  | 0.16 | 0.11 | 0.11 | 0.142 | 0.14 | 0.11 | 0.10 | 0.202 |
| Amotivation PA |  |  |  |  | -0.03 | 0.11 | -0.02 | 0.779 | 0.0003 | 0.11 | 0.0002 | 0.998 |
| Controlled mot. Diet |  |  |  |  | 0.02 | 0.12 | 0.02 | 0.843 | 0.07 | 0.12 | 0.05 | 0.557 |
| Controlled mot. PA |  |  |  |  | -0.10 | 0.12 | -0.07 | 0.410 | -0.14 | 0.12 | -0.10 | 0.269 |
| Autonomous mot. Diet |  |  |  |  | -0.07 | 0.13 | -0.05 | 0.606 | -0.05 | 0.14 | -0.03 | 0.739 |
| Autonomous mot. PA |  |  |  |  | 0.22 | 0.13 | 0.15 | 0.097 | 0.23 | 0.14 | 0.15 | 0.093 |
| Intrinsic mot. Diet |  |  |  |  | 0.16 | 0.12 | 0.09 | 0.158 | 0.19 | 0.12 | 0.10 | 0.113 |
| Intrinsic mot. PA |  |  |  |  | -0.11 | 0.10 | -0.07 | 0.283 | -0.12 | 0.10 | -0.08 | 0.229 |
| Autonomy |  |  |  |  | 0.25 | 0.12 | 0.12 | **0.037** | 0.22 | 0.13 | 0.10 | 0.096 |
| Relatedness |  |  |  |  | 0.34 | 0.18 | 0.17 | 0.055 | 0.31 | 0.18 | 0.16 | 0.078 |
| Competence |  |  |  |  | 0.62 | 0.14 | 0.36 | **<0.001** | 0.59 | 0.14 | 0.34 | **<0.001** |
|  |  |  |  |  |  |  |  |  |  |  |  |  |
| Diet guideline green^f^ |  |  |  |  |  |  |  |  | 1.25 | 1.43 | 0.04 | 0.380 |
| Diet guideline red^f^ |  |  |  |  |  |  |  |  | 0.14 | 0.21 | 0.03 | 0.490 |
| PA guideline green^f^ |  |  |  |  |  |  |  |  | 0.43 | 0.61 | 0.06 | 0.479 |
| PA guideline red^f^ |  |  |  |  |  |  |  |  | -0.32 | 0.73 | -0.04 | 0.660 |
| Module choice Diet^g^ |  |  |  |  |  |  |  |  | -0.45 | 0.31 | -0.11 | 0.141 |
| Module choice PA^g^ |  |  |  |  |  |  |  |  | 0.32 | 0.54 | 0.03 | 0.553 |
| Module choice Both^g^ |  |  |  |  |  |  |  |  | -0.58 | 0.27 | -0.17 | **0.033** |
| Sessions Diet |  |  |  |  |  |  |  |  | 0.03 | 0.07 | 0.04 | 0.610 |
| Sessions PA |  |  |  |  |  |  |  |  | 0.15 | 0.07 | 0.15 | **0.036** |
| Observations | 294 | | | | 291 | | | | 291 | | | |
| R^2^ / R^2^ adjusted | 0.060 / 0.031 | | | | 0.403 / 0.359 | | | | 0.431 / 0.368 | | | |
| AIC | 1162.61 | | | | 1043.14 | | | | 1047.00 | | | |

*Note*. Values in bold represent significance. Results’ interpretations are reported when all other predictors were held constant. When cells in the table are blank, these variables were not included in the model. OR = odds ratio; SE = standard error; mot. = motivation; R^2^ = explained variance; AIC = Akaike information criterion.

^a^ Female is the reference category

^b^ Medium education is the reference category

^c^ Single is the reference category

^d^ No physical impairment is the reference category

^e^ Being unemployed is the reference category

^f^ The orange guideline is the reference category

^g^ No module is the reference category

|  | Appreciation diet | | | | Appreciation diet | | | | Appreciation diet | | | |
| --- | --- | --- | --- | --- | --- | --- | --- | --- | --- | --- | --- | --- |
| *Predictors* | b | SE | B | *P* value | b | SE | B | *P* value | b | SE | B | *P* value |
| Intercept | 6.62 | 1.15 | 0.00 | **<0.001** | 0.89 | 1.05 | 0.00 | 0.401 | 1.17 | 1.35 | 0.00 | 0.386 |
| Age | 0.02 | 0.01 | 0.17 | 0.065 | -0.001 | 0.01 | -0.01 | 0.909 | -0.00 | 0.01 | -0.03 | 0.709 |
| Gender^a^ | -0.15 | 0.30 | -0.04 | 0.626 | -0.15 | 0.19 | -0.04 | 0.454 | -0.15 | 0.20 | -0.04 | 0.448 |
| Education high^b^ | -0.31 | 0.33 | -0.09 | 0.354 | 0.02 | 0.22 | 0.01 | 0.916 | 0.05 | 0.22 | 0.01 | 0.813 |
| Education low^b^ | 0.41 | 0.62 | 0.06 | 0.512 | -0.17 | 0.42 | -0.02 | 0.691 | -0.25 | 0.43 | -0.03 | 0.566 |
| Marital partner^c^ | 0.16 | 0.32 | 0.05 | 0.613 | -0.01 | 0.20 | -0.003 | 0.963 | 0.03 | 0.21 | 0.01 | 0.880 |
| Work^d^ | 0.11 | 0.31 | 0.03 | 0.737 | 0.07 | 0.20 | 0.02 | 0.742 | 0.07 | 0.20 | 0.02 | 0.713 |
| Impairment^e^ | 0.35 | 0.57 | 0.05 | 0.543 | -0.18 | 0.37 | -0.03 | 0.632 | -0.09 | 0.39 | -0.01 | 0.818 |
| BMI | -0.02 | 0.03 | -0.08 | 0.359 | -0.01 | 0.02 | -0.04 | 0.552 | -0.01 | 0.02 | -0.04 | 0.590 |
| Health status | 0.001 | 0.01 | 0.01 | 0.944 | -0.001 | 0.01 | -0.01 | 0.910 | -0.002 | 0.01 | -0.02 | 0.797 |
|  |  |  |  |  |  |  |  |  |  |  |  |  |
| Amotivation Diet |  |  |  |  | -0.08 | 0.11 | -0.06 | 0.452 | -0.10 | 0.11 | -0.07 | 0.383 |
| Amotivation PA |  |  |  |  | 0.20 | 0.11 | 0.15 | 0.072 | 0.24 | 0.12 | 0.17 | **0.047** |
| Controlled mot. Diet |  |  |  |  | -0.10 | 0.15 | -0.07 | 0.510 | -0.09 | 0.15 | -0.07 | 0.534 |
| Controlled mot. PA |  |  |  |  | -0.08 | 0.14 | -0.06 | 0.572 | -0.09 | 0.14 | -0.07 | 0.513 |
| Autonomous mot. Diet |  |  |  |  | 0.23 | 0.17 | 0.16 | 0.179 | 0.25 | 0.19 | 0.17 | 0.181 |
| Autonomous mot. PA |  |  |  |  | 0.09 | 0.18 | 0.07 | 0.591 | 0.09 | 0.19 | 0.06 | 0.643 |
| Intrinsic mot. Diet |  |  |  |  | 0.32 | 0.13 | 0.19 | **0.013** | 0.31 | 0.13 | 0.18 | **0.019** |
| Intrinsic mot. PA |  |  |  |  | -0.09 | 0.11 | -0.06 | 0.380 | -0.09 | 0.11 | -0.06 | 0.401 |
| Autonomy |  |  |  |  | 0.16 | 0.15 | 0.06 | 0.278 | 0.17 | 0.15 | 0.06 | 0.274 |
| Relatedness |  |  |  |  | 0.36 | 0.16 | 0.19 | **0.031** | 0.38 | 0.17 | 0.20 | **0.027** |
| Competence |  |  |  |  | 0.79 | 0.12 | 0.50 | **<0.001** | 0.78 | 0.13 | 0.49 | **<0.001** |
|  |  |  |  |  |  |  |  |  |  |  |  |  |
| Diet guideline red^f^ |  |  |  |  |  |  |  |  | -0.07 | 0.22 | -0.02 | 0.744 |
| PA guideline green^f^ |  |  |  |  |  |  |  |  | 0.04 | 0.68 | 0.01 | 0.956 |
| PA guideline red^f^ |  |  |  |  |  |  |  |  | -0.26 | 0.81 | -0.03 | 0.744 |
| Module choice Diet^g^ |  |  |  |  |  |  |  |  | -0.26 | 0.19 | -0.07 | 0.181 |
| Sessions Diet |  |  |  |  |  |  |  |  | -0.04 | 0.10 | -0.02 | 0.673 |
| R^2^ / R^2^ adjusted | 0.060 / 0.004 | | | | 0.663 / 0.614 | | | | 0.669 / 0.607 | | | |
| AIC | 628.02 | | | | 487.19 | | | | 494.01 | | | |

**Table 2.** Results from the stepwise linear regression predicting the appreciation score of the diet module (*n* = 159)

*Note*. Values in bold represent significance. Results’ interpretations are reported when all other predictors were held constant. When cells in the table are blank, these variables were not included in the model. OR = odds ratio; SE = standard error; mot. = motivation; R^2^ = explained variance; AIC = Akaike information criterion.

^a^ Female is the reference category

^b^ Medium education is the reference category

^c^ Single is the reference category

^d^ No physical impairment is the reference category

^e^ Being unemployed is the reference category

^f^ The orange guideline is the reference category

^g^ Choosing both modules is the reference category

**Table 3.** Results from the stepwise linear regression predicting the appreciation score of the PA module

|  | Appreciation PA | | | | Appreciation PA | | | | Appreciation PA | | | |
| --- | --- | --- | --- | --- | --- | --- | --- | --- | --- | --- | --- | --- |
| *Predictors* | b | SE | B | *P* value | b | SE | B | *P* value | b | SE | B | *P* value |
| Intercept | 7.40 | 1.55 | 0.00 | **<0.001** | 1.82 | 1.34 | 0.00 | 0.176 | 0.36 | 1.63 | 0.00 | 0.826 |
| Age | 0.03 | 0.02 | 0.19 | 0.108 | -0.00 | 0.01 | -0.00 | 0.972 | -0.01 | 0.01 | -0.05 | 0.569 |
| Gender^a^ | 0.10 | 0.38 | 0.03 | 0.797 | 0.30 | 0.26 | 0.09 | 0.237 | 0.19 | 0.27 | 0.05 | 0.490 |
| Education high^b^ | -0.51 | 0.39 | -0.14 | 0.191 | -0.05 | 0.27 | -0.01 | 0.855 | -0.05 | 0.28 | -0.01 | 0.848 |
| Education low^b^ | 1.16 | 1.04 | 0.12 | 0.265 | 0.35 | 0.68 | 0.04 | 0.603 | 0.14 | 0.71 | 0.01 | 0.849 |
| Marital partner^c^ | 0.50 | 0.40 | 0.14 | 0.217 | 0.00 | 0.26 | 0.00 | 0.998 | 0.04 | 0.29 | 0.01 | 0.886 |
| Work^d^ | -0.19 | 0.40 | -0.06 | 0.627 | -0.01 | 0.27 | -0.00 | 0.983 | 0.05 | 0.28 | 0.01 | 0.861 |
| Impairment^e^ | -0.36 | 0.65 | -0.06 | 0.581 | -0.33 | 0.43 | -0.06 | 0.448 | -0.17 | 0.49 | -0.03 | 0.720 |
| BMI | -0.04 | 0.03 | -0.16 | 0.160 | -0.03 | 0.02 | -0.11 | 0.176 | -0.02 | 0.02 | -0.09 | 0.262 |
| Health status | -0.00 | 0.01 | -0.02 | 0.865 | -0.01 | 0.01 | -0.08 | 0.301 | -0.01 | 0.01 | -0.05 | 0.558 |
|  |  |  |  |  |  |  |  |  |  |  |  |  |
| Amotivation Diet |  |  |  |  | -0.27 | 0.17 | -0.18 | 0.119 | -0.37 | 0.18 | -0.24 | **0.048** |
| Amotivation PA |  |  |  |  | 0.23 | 0.16 | 0.16 | 0.167 | 0.34 | 0.17 | 0.23 | 0.058 |
| Controlled mot. Diet |  |  |  |  | -0.06 | 0.16 | -0.05 | 0.701 | 0.02 | 0.17 | 0.02 | 0.901 |
| Controlled mot. PA |  |  |  |  | -0.05 | 0.16 | -0.04 | 0.779 | -0.15 | 0.17 | -0.11 | 0.405 |
| Autonomous mot. Diet |  |  |  |  | -0.06 | 0.18 | -0.04 | 0.734 | -0.10 | 0.19 | -0.07 | 0.606 |
| Autonomous mot. PA |  |  |  |  | 0.43 | 0.19 | 0.31 | **0.025** | 0.52 | 0.20 | 0.37 | **0.013** |
| Intrinsic mot. Diet |  |  |  |  | 0.13 | 0.16 | 0.07 | 0.432 | 0.12 | 0.18 | 0.07 | 0.507 |
| Intrinsic mot. PA |  |  |  |  | -0.06 | 0.14 | -0.04 | 0.684 | -0.04 | 0.15 | -0.03 | 0.816 |
| Autonomy |  |  |  |  | 0.30 | 0.20 | 0.11 | 0.144 | 0.32 | 0.21 | 0.12 | 0.128 |
| Relatedness |  |  |  |  | 0.56 | 0.24 | 0.29 | **0.024** | 0.51 | 0.25 | 0.26 | **0.046** |
| Competence |  |  |  |  | 0.55 | 0.17 | 0.36 | **0.002** | 0.52 | 0.18 | 0.34 | **0.005** |
|  |  |  |  |  |  |  |  |  |  |  |  |  |
| Diet guideline red^f^ |  |  |  |  |  |  |  |  | -0.22 | 0.29 | -0.05 | 0.453 |
| PA guideline green^f^ |  |  |  |  |  |  |  |  | 0.44 | 0.77 | 0.08 | 0.572 |
| PA guideline red^f^ |  |  |  |  |  |  |  |  | 0.54 | 0.88 | 0.09 | 0.540 |
| Module choice Diet^g^ |  |  |  |  |  |  |  |  | -0.14 | 0.51 | -0.02 | 0.793 |
| Module choice PA^g^ |  |  |  |  |  |  |  |  | 0.29 | 0.70 | 0.03 | 0.683 |
| Sessions PA |  |  |  |  |  |  |  |  | 0.27 | 0.16 | 0.13 | 0.093 |
| Observations | 101 | | | | 101 | | | | 101 | | | |
| R^2^ / R^2^ adjusted | 0.126 / 0.039 | | | | 0.710 / 0.637 | | | | 0.726 / 0.630 | | | |
| AIC | 401.28 | | | | 311.98 | | | | 318.10 | | | |

*Note*. Values in bold represent significance. Results’ interpretations are reported when all other predictors were held constant. When cells in the table are blank, these variables were not included in the model. OR = odds ratio; SE = standard error; mot. = motivation; R^2^ = explained variance; AIC = Akaike information criterion.

^a^ Female is the reference category

^b^ Medium education is the reference category

^c^ Single is the reference category

^d^ No physical impairment is the reference category

^e^ Being unemployed is the reference category

^f^ The orange guideline is the reference category

^g^ Choosing both modules is the reference category
